# Federated learning with continual update for privacy-preserving clinical event prediction across distributed hospitals using MCN-GNN

**DOI:** 10.1038/s41598-026-40964-y

**Published:** 2026-03-08

**Authors:** K. Jagdeesh, N. Kanimozhi, Tanvir H. Sardar, N. Naveenkumar, B. Mahalakshmi, A. Chandrasekar, M. Karpagam, Sk Mahmudul Hasan

**Affiliations:** 1 Vel Tech Rangarajan Dr. Sagunthala R&D Institute of Science and Technology, Avadi, Chennai, Tamil Nadu 600062 India; 2 Department of Computational Intelligence, Faculty of Engineering and Technology, SRM Institute of Science and Technology, SRM Nagar, Kattankulathur, Chennai, Tamil Nadu 603203 India; 3 Department of CSE, School of Engineering, Dayananda Sagar University, Bengaluru, 562112 India; 4 Department of Information Technology, Nehru Institute of Technology, Kaliyapuram, Coimbatore, Tamil Nadu 641 105 India; 5 Department of Computer Science and Engineering, M.P.Nachimuthu M.Jaganathan Engineering College, Erode, Tamil Nadu India; 6 Department of Computer Science and Engineering, Nandha College of Technology, Erode, 638052 Tamilnadu India; 7 Manipal Institute of Technology Bengaluru, Manipal Academy of Higher Education, Manipal, 560064 India

**Keywords:** Federated learning, Graph neural network (GNN), Clinical event prediction, Distributed healthcare systems, Medical informatics, Electronic health records, Secure clinical artificial intelligence, Deep learning (DL), Computational biology and bioinformatics, Engineering, Mathematics and computing

## Abstract

Federated Learning (FL) enables accurate and secure Clinical Event Prediction (CEP) across distributed hospitals. However, the prevailing works overlooked the catastrophic forgetting during the global update. Therefore, a Meta Experience Polynomial Decay-based Replay (MEPDR)-centric continual update is proposed. Initially, the hospitals (local model) register and log into the blockchain. Then, to train the CEP model, data collection, pre-processing, and feature extraction are performed. Further, the Temporal-Causal Graph (TCG) is constructed. Afterward, the node matrix is created, and the CEP is done using Mean-Centering Normalization-based Graph Neural Network (MCN-GNN). The model’s gradients are further preserved using the Homomorphic Robust Log Scaling-based Encryption (HRLSE). Next, the hospitals are authenticated using the Exponential Probing Digital Signature Algorithm (ExPrDSA). Thereafter, in the global model, the aggregation is performed using the Calinski–Harabasz Index with Zhonghua Distance-based K-Means Clustering (CHIZD-KMC), followed by global CEP. After that, during the global update, the MEPDR-based continual learning is carried out in each local model. Also, the transactions are stored in the blockchain to enhance traceability. Thus, the proposed system effectively predicted the clinical events with an accuracy of 98.97%, outperforming existing works.

**Supplementary Information:**

The online version contains supplementary material available at 10.1038/s41598-026-40964-y.

## Introduction

The development of Artificial Intelligence (AI), especially in the healthcare system, has transformed a wide range of domains. The healthcare with predictive models helps in the diagnosis and treatment planning^1,2^. In CEP, the Electronic Health Record (EHR) of the patient is analyzed and correlated to improve patient outcomes^3^. However, these healthcare data are often fragmented, thus causing restrictions on centralized data sharing^4^. Therefore, FL has been developed to enable multiple hospitals for collaboratively training the global models without transferring the data of patients^5^. This enables privacy preservation in the distributed healthcare system.

During the deployment of federated learning in real-world environments, the format of the electronic health records and the heterogeneity of the data must be considered^6^. The existing works converted the EHR into an effective format for clinical event prediction. The DL models, like Recurrent Neural Network (RNN) and Long Short-Term Memory (LSTM), were widely used in the prevailing works for clinical event prediction^7,8^. However, the higher-order dependencies couldn’t be captured by these models^9^. Thus, the GNN that represented the clinical events as nodes and relationships as edges was utilized to analyse the patterns in EHR^10^. Some prevailing works integrated GNN with federated learning to provide scalable and privacy-preserving CEP across distributed hospitals.

Nevertheless, the traditional FL-based clinical event prediction models exhibited significant limitations^11^. The prevailing models shared the gradients without privacy preservation into the global model. This resulted in inference of attacks and improper CEP^12^. Also, the global model’s performance was degraded due to the participation of malicious hospitals in the distributed hospital network^13^. In the prevailing works, the coordination between diverse participants became complex due to the absence of transparent records^14^. Furthermore, adapting to the shared knowledge without losing the local credits remained a challenge^15^. The motive of the paper is to safeguard the sensitive patient information and resist the adversarial interference with precise FL-based CEP. Hence, a novel MEPDR-based continual update and MCN-GNN-based CEP is proposed.

### Problem statement

The drawbacks present in the prevailing works are described as follows,

- None of the prevailing works overcame the catastrophic forgetting, thus resulting in a drop in performance in predicting the clinical events after the global update.
- In the existing^16^, sharing model gradients without encryption caused attackers to exploit the patterns through model inversion attacks, leading to serious privacy risks.
- In the prevailing^17^, FL was vulnerable to malicious hospitals that send adversarial model updates, thus degrading the global model’s performance and reliability.
- Many hospitals distrusted centralized aggregation servers due to a lack of transparent and verifiable records of participation^18^.
- In prevailing^19^, directly aggregating updates from heterogeneous local models led to misalignment and unreliable global model updates.
- Most of the existing models learned patterns based on connections and correlations in data rather than true causal relationships. Thus, the performance of the prediction was reduced.

*Core Contribution of the Proposed Work:* Here, the proposed work develops a federated learning with continual update for privacy-preserving clinical event prediction across distributed hospitals. Also, the proposed work addresses catastrophic forgetting in distributed hospital settings through MEPDR, ensuring continual learning without loss of local expertise. Further, the prediction accuracy is enhanced by constructing the Temporal causal graphs and using the MCN-GNN while mitigating over-smoothing. Moreover, the privacy during model gradient updation is preserved using the HRLSE, whereas the hospital authentication is strengthened using the ExPrDSA. Further, to improve aggregation reliability, the CHIZD-KM is applied for cluster-wise updates. Finally, blockchain integration confirms transparent, immutable traceability of all transactions, making the system secure across distributed hospitals.

### Objectives

The contributions of the proposed approach are given below,

- The MEPDR-based continual update is provided to the local model during the global update. This helps in overcoming the catastrophic forgetting issue and increases the prediction performance.
- For the privacy preservation of the local model’s output gradients, the HRLSE method is utilized. Thus, the model inversion attack is avoided.
- To avoid adversarial model updates and to enhance the global model’s performance, the hospitals are authenticated using ExPrDSA.
- The transparent and verifiable record of participation with fairness and accountability is provided by storing all the transactions in the blockchain.
- To aggregate the heterogeneous data from the local models into the global model, the cluster-wise aggregation is performed using the CHIZD-KMC approach.
- For learning the causal relationship and the correlation of the electronic health records data, the TCG is constructed and further utilized for MCN-GNN-based clinical event prediction.

The paper is structured as: The related works are explained in Section "Literature survey", the proposed method is described in Section "Proposed methodology for privacy-preserving clinical event prediction via federated learning", the performance of the proposed system is analyzed in Section "Results and discussion", and lastly, the paper is winded up in Section "Conclusion" with future scope.

## Literature survey

Meduri et al.^16^ presented FL for privacy-preserving of the EHR in clinical research. The EHR for multiple institutions was collected. Then, the classifier models, such as Logistic Regression, Support-Vectors-Classifiers, Decision-Tree-Classifiers, Random-Forests, and Stacking-Classifiers, were utilized for disease diagnosis. Here, the FL was used to enhance privacy preservation. Thus, the disease was effectively detected. However, the sensitive information was not preserved, thus leading to serious privacy risks during data sharing. Also, Messinis et al.^17^ established private client selection and resource allocation in FL for medical applications. Here, the medical information of the patients was collected from the distributed hospitals. Then, the GNN was utilized for the detection of clinical events. Next, the aggregation of the data into the local model was carried out by client selection. Hence, the CEP with privacy was maintained. Nevertheless, the hospitals were not authenticated. Thus, the malicious hospitals sent adversarial model updates, degrading the model’s performance. Furthermore, Ahmed et al.^18^ developed an FL model with dynamic scoring-centric client selection for disease diagnosis. At first, the server was initialized, and the local training was carried out using a random forest. Then, the output of the model was aggregated based on the dynamic score. Further, the global model was trained centered on the aggregated data in the FL. Thus, the disease was precisely diagnosed. On the other hand, the model lacked transparent and verifiable records of participation, thereby reducing the reliability of disease prediction. Similarly, Nagamani et al.^19^ estimated a graph-centric model for anomaly detection in healthcare using FL. Here, the EHR data was collected from the patients. Then, the data was integrated using Kafka, and the real-time processing was carried out. Further, the local model was trained using the Convolutional Neural Network and LSTM (CNN-LSTM). Then, the gradients were aggregated, and the global model was updated. Afterward, based on multimodal attention, the anomaly score was predicted; then, the alert was generated effectively. But, the direct aggregation led to misalignment of global model updates. Additionally, Ali et al.^20^ introduced a privacy-preserving FL framework for scalable and secure healthcare investigation. Here, the healthcare data was collected from the data owner and stored in each hospital. Next, the Homomorphic Encryption (HE) was applied to each local model’s data. Then, the security was validated in the homomorphic computation unit, and the regulatory compliance was also applied. Next, the large-scale data was transferred into the global model for further processing. Hence, the privacy of the healthcare data was effectively preserved. Yet, the gradient stability of the model was improper, thus degrading the local update.

Moreover, Kuliha and Verma^21^ explored the security of EHR using the trusted decentralized FL consensus mechanism. The doctor and patient logged into the blockchain. After that, the EHRs of the patients were collected, and the missing values were imputed. Subsequently, the data were normalized utilizing the min–max technique. Thereafter, the features were selected. By utilizing a Generative Adversarial Network (GAN), the disease was predicted. These gradients were further transferred to the cloud server that had a local model. Thus, the prediction of the disease was precisely carried out. However, due to catastrophic forgetting, the local model couldn’t predict the disease, leading to a performance drop. Also, Zhao et al.^22^ demonstrated a multi-source EHR prognosis identification via a privacy-preserved FL approach. Here, a privacy-aware multi-channel architecture securely embedded every single clinical feature separately in clinical representation learning. This framework allowed every individual to maintain their sensitive clinical information, showing high reliability. But, this model had considerable latency in model updates aggregation, thereby resulting in outdated global models. Further, Abaoud et al.^23^ utilized an advanced framework named FL-based privacy preservation in healthcare systems. Here, a privacy-preserving methodology like a secure multi-differential privacy model was employed to ensure data privacy. Then, the secured data was analyzed, and the gradients were transferred to the server. Thus, the privacy was preserved, and the diagnosis was carried out. However, the cause-and-effect relationship was not analysed, thus reducing the diagnosis performance. Next, Akter et al.^24^ employed a serverless privacy edge intelligence-centric federated learning model in smart healthcare systems. In this work, the federated edge aggregator and authentication methodology were included to enhance data privacy and enable client adaptation. Here, the model classified the intruders via serverless computing processes. Hence, it attained higher accuracy and provided enhanced medical security with serverless computing. Nevertheless, the model increased the processing delay. Likewise, Edelson et al.^25^ introduced a framework for false-positive-tolerant misconduct mitigation in distributed federated learning in clinical institutions. Here, the false-positive tolerant methodology was established for preserving model integrity and mitigating the effects of adversarial misconduct in FL. The model prevented over-ostracization and the subsequent loss of sample size. However, the model still had issues with heterogeneous data quality and varying record-keeping standards across clinical institutions.

Additionally, Sharma et al.^26^ implemented a medical diagnosis model using Graph Neural Networks (GNN) for medical images. At first, the medical images were segmented into regions of interest and further normalized. Next, they were represented as a graph and fed into a structural GNN. Finally, a multi-task learning approach was employed within the GNN to handle disease classification and severity prediction. As a result, the model attained 92.27% Area Under the Curve (AUC). Nevertheless, the model suffered from noise sensitivity and over-smoothing limitations, causing reduced learning efficiency. Similarly, Bi et al.^27^ presented a Federated-Decentralized-Learning Graph Attention Network for Doctor Recommendation (FD-GATDR) model using Electronic Health Records (EHRs). The model utilized Bi-directional Encoder Representations from Transformers-Long Short-Term Memory (BERT-LSTM) for service code embedding. Then, the Heterogeneous Graph Attention Network (HGAT) was utilized to learn structured representations from EHR data. Further, Federated Decentralized Learning (FDL) handled decentralized data. Hence, the model improved AUC by up to 6.2%. However, the model had low communication efficiency when handling complex heterogeneous graph structures. Also, Saemaldahr and Ilyas^28^ developed a patient-centric preictal pattern-aware epileptic seizure detection based on federated learning. The spiking encoder with a graph convolutional neural network served as the local model, which was trained using a bi-timescale approach. Centered on the federated learning outcomes, the adaptive neuro-fuzzy inference system was used to identify the epileptic seizure patients. Here, the three-tier architecture for epileptic seizure prediction was presented to improve the model’s learning capability while maintaining data privacy. This model offered fine-grained personalization. However, this framework struggled to handle the high dynamics of epileptic EEG signals, causing classification errors. Finally, Mao et al.^29^ propounded an integrated federated learning with a split learning framework for brain disease prediction using a spatio-temporal graph network. Here, federated learning and spatial learning techniques were applied. A time-aware scheme was applied in the client temporal model to capture the functional changes in the brain structure. In the server spatial model, a graph convolutional neural network was integrated with federated learning. This model significantly improved the diagnostic efficiency through a federated learning scheme. But, this scheme had considerable latency owing to the complex architecture.

Overall, the existing federated learning and GNN-based healthcare models suffer from key limitations, such as weak gradient privacy, lack of authentication, over-smoothing in graph learning, catastrophic forgetting in continual updates, high communication latency, and absence of verifiable audit trails. Therefore, by integrating the MCN-GNN, HRLSE, ExPrDSA, and MEPDR with the proposed work, the proposed work effectively addresses the above mentioned limitations and provides high accuracy during clinical event prediction. Also, the proposed work ensures security, stability, and scalability in federated clinical event prediction compared to traditional works.

## Proposed methodology for privacy-preserving clinical event prediction via federated learning

**Fig. 1:**
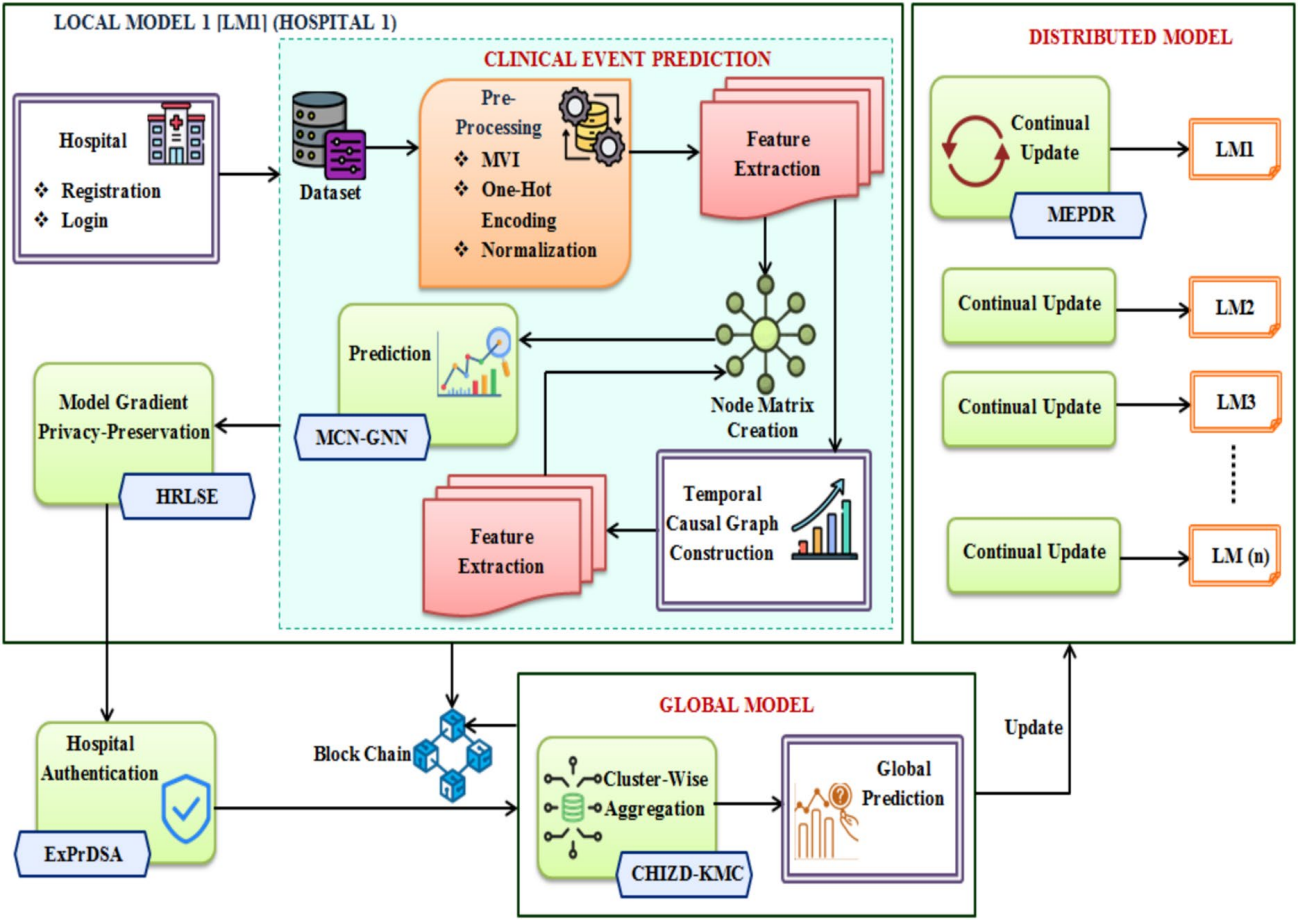
Architecture of the proposed framework.

In the proposed work, privacy is preserved, and the FL-based clinical event prediction is carried out regarding the EHR. The important steps involved in the proposed framework are TCG construction, CEP, model gradient privacy-preservation, hospital authentication, cluster-wise aggregation, and continual update in the local model. The structure of the proposed system is demonstrated in Fig. 1.

In the proposed work, the proposed MCN-GNN is introduced for excellently performing CEP. Also, the proposed HRLSE is employed to secure the output gradient of the local model. For avoiding the adversarial model updates, the hospital authentication is performed using the proposed ExPrDSA. Likewise, the proposed CHIZD-KMC is employed to aggregate the heterogeneous data from the local models into the global model. To avoid the catastrophic forgetting issue, increase the prediction performance, and eliminate the overfitting issue, the proposed MEPDR is used for continual update during global update.

In the proposed FL system, there are a $\left( n \right)$ number of local models, which act as distributed hospitals $\left( H \right)$. All these $\left( H \right)$ are heterogeneous, i.e., some models predict stroke, some diagnose heart failure, and so on. Here, the $\left( n \right)$ number of local models is trained for CEP regarding heart failure EHR, stroke data, and cirrhosis prediction data. Eventually, all the trained local models are updated in the global model.

### Local model 1

In this proposed approach, the local model 1 is trained for clinical event prediction based on the heart failure EHR. The processes carried out in the local model 1 are explained in the following sections.

#### Hospital registration and login

At first, the registration of Local Model 1 (Hospital 1) $\left( {H_{1} } \right)$ into the blockchain is carried out. Here, due to the nature of the blockchain, such as decentralisation, immutability, and transparency, each hospital’s identity and registration details are securely recorded, preventing unauthorised access and tampering. However, without blockchain, the registration process relies on a centralised server, which is vulnerable to single-point failures, unauthorised access, and data tampering. Also, the blockchain layer functions as a thin trust mechanism, making the architecture both secure and feasible for real-world multi-hospital deployments. Therefore, the hospital registration is carried out on the blockchain network. In this, the registration and login of $\left( {H_{1} } \right)$ are done based on the hospital Identification (ID) and password. Further, the CEP is performed.

#### Clinical event prediction

Here, to predict heart failure, the clinical event prediction model is trained. For training, the data collection, pre-processing, feature extraction, TCG construction, and prediction are done. The step-by-step process for training the CEP model is described as follows,

##### Dataset

The heart failure prediction dataset, which consists of EHR, has been collected. The data $\left( E \right)$ from this dataset is utilized for the training of $\left( {H_{1} } \right)$. Here, the total $\left( h \right)$ number of data is available. Next, $\left( E \right)$ is pre-processed.

##### Pre-processing

Thereafter, to convert $\left( E \right)$ into a structured format and to ensure reliable analysis of the data, the pre-processing steps, namely Missing Value Imputation (MVI), one-hot encoding, and normalization, are done.

- Missing Value Imputation

Primarily, the missing values in $\left( E \right)$ are filled using the MVI process. Here, the mean of the neighbouring value in the dataset is used for imputing the missing data. By filling in the missing values, the data is made compatible. Let the MVI output be represented as $\left( {E^{\prime\prime}} \right)$.

- On-Hot Encoding

Afterward, the categorical variables present in $\left( {E^{\prime\prime}} \right)$ are converted into a binary vector using One-Hot Encoding (OHE). This process identifies the categorical variables in $\left( {E^{\prime\prime}} \right)$ and assigns a binary vector for each category. Thus, the DL models efficiently process the data. The output of OHE is signified as $\left( {\dddot E} \right)$.

- Normalizatio***n***

Then, $\left( {\dddot E} \right)$ is normalized in the range of 0 to 1 using the min–max normalization technique, which is provided in Eq. (1). This helps in preserving the original data distribution and trains the CEP model precisely.

1 $$\tilde{E} = \frac{{\dddot E - \dddot E^{\min } }}{{\dddot E^{\max } - \dddot E^{\min } }}$$

Here, $\left( {\tilde{E}} \right)$ is the normalized output,$\left( {\dddot E^{\min } ,\dddot E^{\max } } \right)$ are the minimum and maximum values of $\left( {\dddot E} \right)$, respectively, and $\left( {\tilde{E}} \right)$ is the final pre-processed data. After that, features are extracted from $\left( {\tilde{E}} \right)$ for further analysis.

##### Feature extraction

Next, from $\left( {\tilde{E}} \right)$, the features, namely age, resting blood pressure, sex, chest pain type, cholesterol level, resting electrocardiogram results, maximum heart rate achieved, old peak, fasting blood sugar, exercise-induced angina, and so on, are extracted. The extracted features are denoted as $\left( F \right)$. Further, the TCG is constructed.

##### Temporal-causal graph construction and feature extraction

In this section, to capture the cause-and-effect relationships between clinical events over time in $\left( F \right)$, the TCG is constructed. TCG excellently provides differentiation of true cause and effect relationships from mere correlations. The TCG analyzes whether earlier event data influences the later one or not. The features $\left( F \right)$ act as nodes (events)$\left( y \right)$, and the links with temporal constraints $\left( g \right)$ serve as edges $\left( z \right)$. Next, for capturing the temporal influences, lagged vectors are generated to a maximum lag $\left( {\ell g} \right)$. Afterward, the temporal causal relationship is analyzed for identifying how the variable relies on its past values and its causal parent values change at earlier times.

2 $$\varepsilon \varpi_{ab \to mn} = \frac{1}{nT - 1}\sum\limits_{tm = \ell g + 1}^{nT} {y_{ab} \left( {tm - \ell g} \right)y_{mn} \left( {tm} \right)}$$

where, $\varepsilon \varpi_{ab \to mn}$ specifies the edge weight, $nT$ indicates the number of time steps $\left( {tm} \right)$, and $y_{ab}$ and $y_{mn}$ signify the $ab^{th}$ and $mn^{th}$ nodes, correspondingly. Then, the $\varepsilon \varpi_{ab \to mn}$ are normalized to prevent the relative causal influence, and it is represented as $\overline{\varepsilon \varpi }_{ab \to mn}$. Thereafter, edge thresholding is carried out. Next, the temporal causal adjacency matrix $\left( {\alpha d_{mnab} } \right)$ is defined as,

3 $$\alpha d_{mnab} = \left\{ {\begin{array}{*{20}c} {{\mathrm{if}}\;\;er_{ab \to mn} \in z} & {\overline{\varepsilon \varpi }_{ab \to mn} } \\ {{\mathrm{otherwise}}} & 0 \\ \end{array} } \right.$$

Here, $er_{ab \to mn}$ denotes the directed edges. Also, node-level connectivity strength is analyzed. Thus, the constructed TCG $\left( \Im \right)$ is given in Eq. (4) as,

4 $$\Im = \left[ {F,y,z,\varepsilon \varpi _{{ab \to mn}} } \right]$$

Then, from $\left( \Im \right)$, features are extracted. The TCG-based features $\left( N \right)$, like in-degree, out-degree, weighted degree, edge weights, statistical confidence of causal links, strongly connected components, causal chain lengths, temporal motifs, node, and so on, are extracted from $\left( \Im \right)$. Here, the total $\left( l \right)$ number of TCG-based features exists. Further, the node matrix is created.

##### Node matrix creation

Subsequently, the node matrix $\left( K \right)$ is created based on the features $\left( {F,N} \right)$ from pre-processed data and TCG, respectively. This $\left( K \right)$ is given as input to the proposed CEP classifier.

5 $$K = \left( {F,N} \right)\,H_{1}$$

Afterward, $\left( K \right)$ is fed into the MCN-GNN classifier.

##### Prediction

**Fig. 2:**
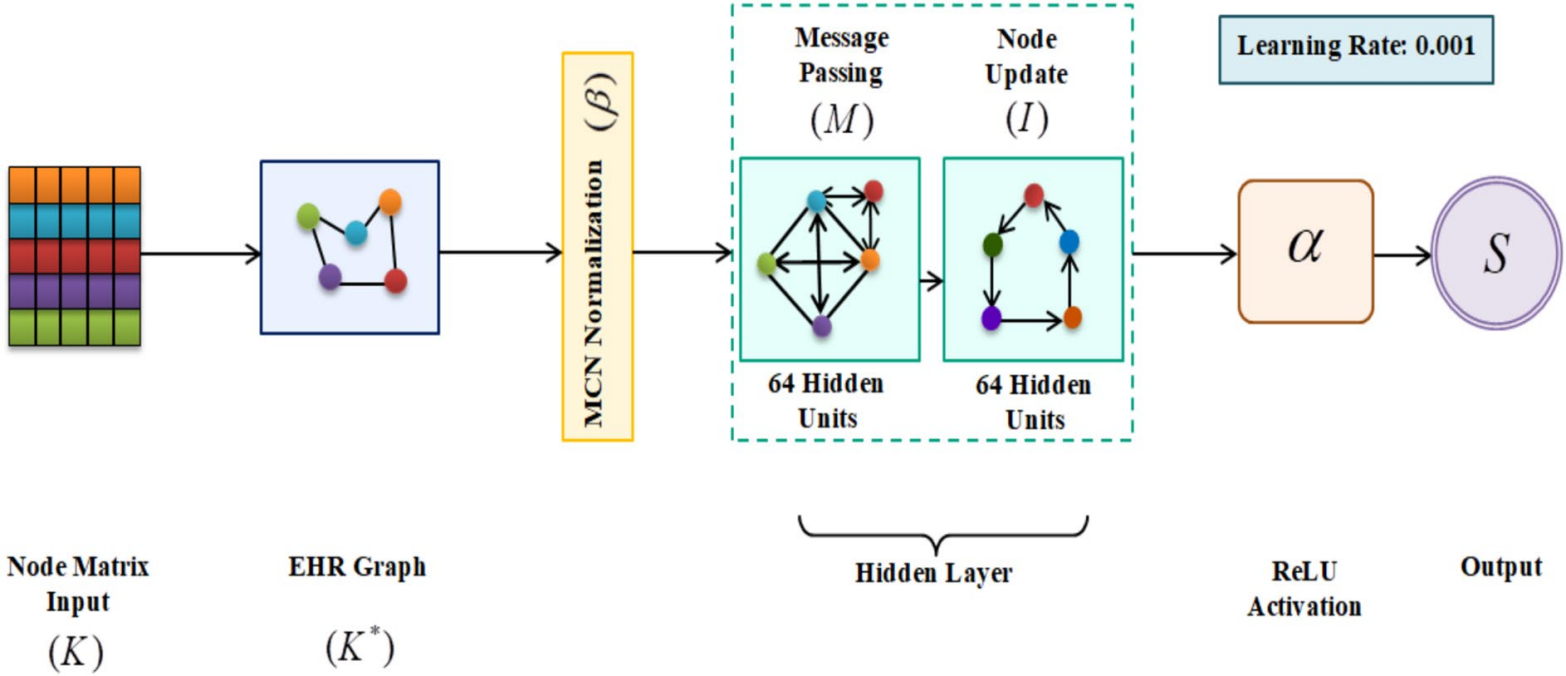
MCN-GNN classifier.

In this phase, the clinical event is predicted based on the created node matrix $\left( K \right)$ using MCN-GNN. The GNN that captures complex relationships between nodes (clinical events) and edges (features) for large-scale information is used for heart failure CEP. It also captures the higher-order dependencies across the entire EHR of a patient. Also, it has excellent parameter sharing and strong inductive capabilities. Normally, a GNN consists of two layers, such as message passing and node update. Yet, the GNN has an over-smoothing issue, i.e., all nodes tend to become indistinguishable from each other after several layers of message passing, thus reducing the discrimination and classification performance. Therefore, various smoothing approaches like Min–Max Scaling, Z-Score Normalization, and Mean-Centering Normalization (MCN) are supported for node stabilization from over-smoothing. Among them, MCN is considered as the best function since Min–Max Scaling and Z-Score Normalization handle either mean shift or scale, leading to ineffective preservation of node-level distinctions across layers. MCN diminishes the excessive feature averaging and improves the robustness by preserving node-specific information, ensuring that each clinical event retains its unique representation. Also, MCN enhance the GNN’s ability to capture higher-order dependencies across the entire HER without losing discriminative power, thus improving the classification accuracy of heart failure prediction. The architecture of the MCN-GNN classifier is depicted in Fig. 2.

- EHR Graph

The node matrix $\left( K \right)$ is fed into the MCN-GNN model. Primarily, $\left( K \right)$ is utilized to generate the EHR graph $\left( {K^{ * } } \right)$. Here, $\left( {K^{ * } } \right)$ is constructed to model the relationship of the features (nodes) across time $\left( t \right)$. Also, $\left( {K^{ * } } \right)$ helps the model to learn correlations and interaction patterns within patient health information.

6 $$K^{ * } = \left[ K \right] \times t$$

Then, the reduction of node complexity is performed to keep the most informative clinical features.

- Normalization

Then, to prevent over-smoothing and to differentiate the nodes with less complexity, the MCN is applied to $\left( {K^{ * } } \right)$. The MCN reduces the drift across layers and preserves all the individual node features. The MCN output $\left( \beta \right)$ is expressed in Eq. (7) as,

7 $$\beta = \left\langle {\frac{{K_{1}^{ * } + K_{2}^{ * } }}{2}} \right\rangle * K^{ * }$$

Here, $\left( {K_{1}^{ * } ,K_{2}^{ * } } \right)$ are the two nodes of $\left( {K^{ * } } \right)$. Next, the processing of $\left( \beta \right)$ is carried out in the hidden layer of the proposed classifier.

- Hidden Layer

In the hidden layer, the message passing and the node update are carried out. This helps in capturing the higher-order dependencies in EHR data. Also, the hidden layer effectively learns complex clinical relationships.

***Message Passing***

Initially, the message passing $\left( M \right)$, which is said to be neighbourhood aggregation, is performed. Each node in $\left( \beta \right)$ collects information from its connected neighbours and analyzes the cause-and-effect and correlation between the input data.

8 $$M = \left\{ {\left[ {\left[ {M*\chi } \right] + \delta } \right]} \right\} \times \alpha$$

9 $$\alpha = \max \left( {0,\beta } \right)$$

where, $\left( {\chi ,\delta } \right)$ are the weight and bias values of $\left( \beta \right)$, correspondingly, and $\left( \alpha \right)$ is the Rectified Linear Unit (ReLU) activation function that introduces non-linearity and learns complicated patterns and relationships within the input data.

***Node Update***

After that, the aggregated data $\left( M \right)$ is combined with the node’s current state to produce a new state that encodes global contextual knowledge and local feature information.

10 $$I = \beta + M$$

Here, the node update output is represented as $\left( I \right)$, which is provided in Eq. (10).

- ReLU Activation

Further, the ReLU activation is done in the readout layer. Here, the summation of the nodes and activation is done to represent the data in a single vector $\left( U \right)$. ReLU improves non-linear separability and suppresses inappropriate negative activations.

11 $$U = \left\{ {\left[ {\sum I *\chi } \right] + \delta } \right\}*\alpha$$

Thus, the final heart failure CEP output $\left( S \right)$ is predicted in the feed-forward layer. The prediction output of the local model 1 (Hospital 1) is depicted as,

12 $$S = \alpha \times \left\langle {\left[ {U*\chi } \right] + \delta } \right\rangle$$

13 $$S = \left\langle {S_{1} ,S_{2} } \right\rangle$$

where, $\left( {S_{1} ,S_{2} } \right)$ are the normal and heart disease classes, respectively. Hence, the clinical event prediction of the local model 1 is carried out. The pseudocode for MCN-GNN is given as follows,


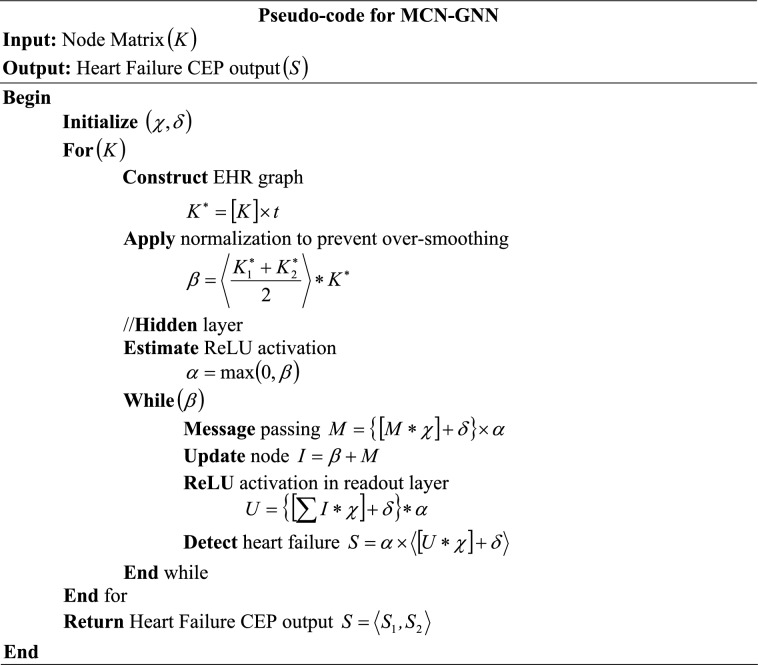


In real time, when the EHR data is entered by Hospital 1 $\left( {H_{1} } \right)$, the heart failure of the patient is predicted. Next, the gradients $\left( R \right)$ of $\left( S \right)$ are preserved.

#### Model gradient privacy preservation

Afterward, to secure the output gradient of the local model 1, the HRLSE is used. The traditional lightweight encryption techniques like Elliptic Curve Cryptography (ECC), HE, Rivest-Shamir-Adleman (RSA), and Advanced Encryption Standard (AES) are well-suited for enhancing data security and privacy preservation. Amongst them, the HE is a powerful cryptographic model that significantly reduces the risk of data leakage while preserving sensitive information. Also, it enables computations to be performed directly on encrypted data without the need for decryption. The HE helps to prevent model inversion attacks by encrypting the model’s gradient $\left( R \right)$, thus ensuring that the data is never exposed. The attackers can’t directly inspect the data mapping, making it impossible to infer the model’s gradient. On the contrary, noise is known as a random error that is added to ciphertexts during encryption, thereby making the distributions indistinguishable. Therefore, various scaling models like Log Scaling, Box-Cox Transform, and Robust Log Scaling (RLS) help to control the noise growth. Among these, the RLS is selected as the log scaling and box-cox transform approaches are sensitive to outliers. RLS avoids the noise growth produced by continual homomorphic additions and multiplications. Also, by integrating RLS with HE, the proposed HRLSE achieves efficient and accurate gradient encryption while preventing excessive noise accumulation during repeated homomorphic operations.

Initially, to avoid the accumulation of noise during privacy preservation, the RLS that limits the amplification of noise and maintains features distinctness is applied to $\left( R \right)$. By scaling $\left( R \right)$, the large gradient magnitudes are compressed into a controlled range, thereby mitigating the noise or outlier accumulation. The RLS output $\left( {\mathop{R}\limits^{\leftrightarrow} } \right)$ is expressed in Eq. (14) as,

14 $$\mathop{R}\limits^{\leftrightarrow} = R * \log \,\left[ {\frac{\left| R \right|}{q} + 1} \right]$$

where, ${\mathrm{log}}$ implies the logarithmic function and $\left( q \right)$ is the constant for numerical stability. Next, the public key $\left( \varepsilon \right)$ and private key $\left( \phi \right)$ utilized for the encryption of the input data $\left( {\mathop{R}\limits^{\leftrightarrow} } \right)$ are generated.

15 $$\varepsilon = \,\mathop{R}\limits^{\leftrightarrow} + \left( {d,e} \right)$$

16 $$\phi = \varepsilon + \mathop{R}\limits^{\leftrightarrow}$$

Here, $\left( {d,e} \right)$ are the noise parameters. Finally, to avoid the model inversion attack and loss of gradient information, the encryption is performed regarding $\left( \varepsilon \right)$ and $\left( \phi \right)$. The privacy-preserved gradient of the local model 1 $\left( G \right)$ is given in Eq. (17) as,

17 $$G = \mathop{R}\limits^{\leftrightarrow} \left( {d,e} \right) + \varepsilon + \phi$$

Hence, in the FL system, the model’s output is secured throughout the prediction process. Thereafter, Hospital 1 is authenticated. Similarly, the Local model 2 is trained based on the stroke prediction dataset for identifying whether the patient has a stroke or not. Also, the Local model 3 is trained regarding the cirrhosis prediction dataset for detecting cirrhosis disease (liver disease). Likewise, all the $\left( n \right)$ number of local models are trained and finally updated in the global model.

### Hospital authentication

Next, $\left( G \right)$ is transferred to the global model of the FL system. Before transferring $\left( G \right)$, Hospital 1 $\left( {H_{1} } \right)$ is authenticated using ExPrDSA. The Digital Signature Algorithm (DSA) is relatively faster in generating a large number of digital signatures, thus rapidly performing the authentication. Also, DSA ensures that the information has not been tampered with during transmission. However, the hash function used by the DSA for generating signatures can be hacked, resulting in information loss and improper authentication. Thus, in DSA, the hash function is generated with the help of linear activations, sigmoid activations, or the Exponential Probing (ExPr) function. Here, the linear and sigmoid actions are vulnerable to collision or hash-flooding attacks, affecting the authentication robustness. Therefore, the ExPr is applied to generate a hash function, which makes collision prediction harder and improves the robustness against hash-flooding attacks; also, it avoids information loss. Additionally, by incorporating the ExPr function with the DSA, the proposed ExPrDSA becomes more resilient to cryptographic attacks, thereby ensuring that digital signatures remain secure even under adversarial conditions.

Primarily, the digital signature is created based on the Hospital ID $\left( T \right)$, public key $\left( \varepsilon \right)$, and the hash function $\left( \gamma \right)$. As explained in Section "Model gradient privacy preservation", the public key $\left( \varepsilon \right)$ is generated. Moreover, to avoid hacking, a hash function $\left( \gamma \right)$ is generated using the ExPr function, which makes the hash less predictable and harder to exploit. Likewise, the ExPr function allows enhanced resistance to adversarial collisions.

18 $$\gamma \left( {H_{1} } \right) = \left( u \right) + \exp \left\{ {\,\bmod \left[ {T\left( {H_{1} } \right)} \right]\,} \right\}$$

Here, $\left( u \right)$ is the constant value, $\left( {\exp } \right)$ is the exponential function, and $\left( {\bmod } \right)$ is the modulus operator. Based on $\left( \gamma \right)$, $\left( T \right)$, and $\left( \varepsilon \right)$, the digital signature $D\left( {H_{1} } \right)$ is created for hospital 1.

19 $$D\left( {H_{1} } \right) = T\left( {H_{1} } \right) + \gamma \left( {H_{1} } \right) + \varepsilon$$

This $D\left( {H_{1} } \right)$ is given to the global model for authentication. After that, the global model $\left( Q \right)$ generates $\gamma \left( Q \right)$ using $G\left( {H_{1} } \right)$.

20 $$\gamma \left( Q \right) = \left( u \right) + \exp \left\{ {\,\bmod \left[ {T\left( {H_{1} } \right)} \right]\,} \right\}$$

Further, the digital signature $D\left( Q \right)$ at the global model is generated using $\left( T \right)$, $\left( \varepsilon \right)$, and $\gamma \left( Q \right)$.

21 $$D\left( Q \right) = T\left( {H_{1} } \right) + \gamma \left( Q \right) + \varepsilon$$

Thereafter, the digital signature verification is done regarding $D\left( {H_{1} } \right)$ and $D\left( Q \right)$ for the authentication of hospital 1 $\left( {H_{1} } \right)$ and to transmit the privacy-preserved gradient $\left( G \right)$ into $\left( Q \right)$. The hospital authentication output $\left( J \right)$ is given in Eq. (22) as,

22 $$J = \left\{ {\begin{array}{*{20}c} {if\;D\left( {H_{1} } \right) = D\left( Q \right)} & {J^{1} } \\ {if\;D\left( {H_{1} } \right) \ne D\left( Q \right)} & {J^{2} } \\ \end{array} } \right.$$

where, $\left( {J^{1} ,J^{2} } \right)$ are the hospital authenticated and non-authenticated outputs, respectively. The condition states that if the generated digital signatures on the hospital and global model sides are equal, then $\left( {H_{1} } \right)$ is said to be authenticated $\left( {J^{1} } \right)$. If the digital signatures are not equal, then $\left( {H_{1} } \right)$ is said to be non-authenticated $\left( {J^{2} } \right)$. The pseudo-code for ExPrDSA is given as follows,


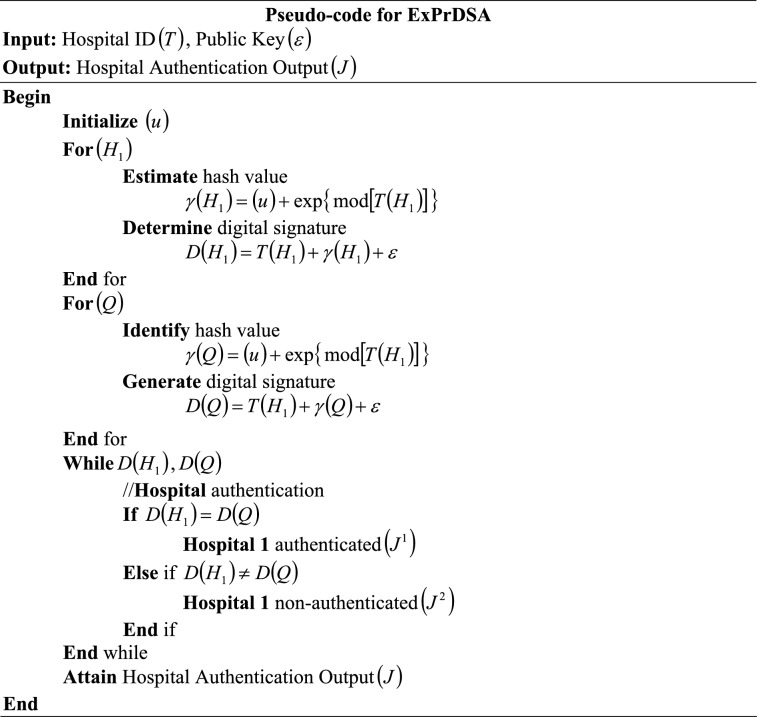


Thus, if $\left( {J^{1} } \right)$ is attained, then the privacy-preserved gradient $\left( G \right)$ is transmitted to $\left( Q \right)$; if $\left( {J^{2} } \right)$ is attained, then the transmission of $\left( G \right)$ is blocked. Hence, the authentication of hospital 1 is carried out effectively. Further, the aggregation of gradients from all the local models is done.

### Global model

In this phase, the gradients from the authenticated heterogeneous hospitals $J^{1} \left( H \right)$ are collected and aggregated. Then, the global prediction, which is the main part of the FL, is done.

#### Cluster-wise aggregation

Initially, the cluster-wise aggregation of the gradient $\left( G \right)$ related to different disease diagnosis outputs from $J^{1} \left( H \right)$ is performed using the CHIZD-KMC approach. Here, the K-Means Clustering (KMC) that automatically partitions the participating hospitals into clusters based on the similarity of their local model gradient $\left( G \right)$ is used for aggregation. This enables cluster-wise aggregation in the global model and ensures that updates from hospitals with similar data distributions are aggregated together to improve convergence. However, KMC is sensitive to the initial placement of centroids, leading to inconsistent results. Moreover, diverse initializations can cause the KMC to converge to different local minima. Additionally, the reliance on Euclidean distance misrepresents the data and fails to capture the true structure of the data, causing suboptimal clustering and poor cluster formation. Among the various centroid initialization techniques, like Silhouette index, Dunn index, and Calinski–Harabasz Index (CHI), the CHI is effective because the silhouette and Dunn indexes are sensitive to the cluster shapes, affecting the overall performance. Therefore, the CHI, which encourages initial centroid placement by maximizing the ratio of between-cluster dispersion to within-cluster dispersion, is used. Also, in KMC, the Zhonghua Distance (ZD) is used instead of Euclidean distance, Manhattan distance, or Cosine Similarity. Here, the Euclidean, Manhattan, or cosine demonstrate poor performance in non-linear and high-dimensional spaces. Hence, the Zhonghua Distance (ZD) is applied to compute the distance by integrating distribution-aware deviations between data points and centroids; also, ZD diminishes the sensitivity to noise and outliers. Further, the proposed CHIZD-KMC achieves more consistent and robust clustering of participating hospitals, ensuring that hospitals with similar local model gradients are grouped accurately using the CHI-based centroid initialisation and ZD-based distance measurement.

Let the gradients $\left( G \right)$ attained from $J^{1} \left( H \right)$ be represented in Eq. (23) as,

23 $$G = \left\{ {G\left[ {J^{1} \left( {H_{1} } \right),J^{1} \left( {H_{2} } \right),J^{1} \left( {H_{3} } \right),J^{1} \left( {H_{4} } \right),....,J^{1} \left( {H_{n} } \right)} \right]} \right\}$$

Here, $\left( n \right)$ is the number of distributed hospitals. Next, the centroid $\left( L \right)$ from the total gradients $\left( G \right)$ attained is selected using the CHI, which provides a clear numerical measure of cluster quality and diminishes unnecessary iterations.

24 $$L = \frac{{\eta^{1} }}{{\eta^{2} }} * \left[ {\frac{n\left( G \right) - p}{{p - 1}}} \right]$$

Here, $\left( {\eta^{1} ,\eta^{2} } \right)$ are the dispersion matrix traces between the clusters and within the clusters, respectively, and $\left( p \right)$ is the number of clusters. This centroid expresses a similar data distribution across the heterogeneous hospitals. Thus, the $vs$ number of initialized centroids is represented as $L\,$. Subsequently, the ZD that combines the distribution similarity and geometric proximity is estimated. ZD ensures a more robust and adaptive measure of similarity. This distance is measured between $\left( L \right)$ and the gradient from the authenticated hospital $J^{1} \left[ {G\left( H \right)} \right]$.

25 $$V = \sqrt {\sum {r * \kappa \left( {L - J^{1} \left[ {G\left( H \right)} \right]} \right)} } * \left\{ {1 + \vartheta \left( {L,J^{1} \left[ {G\left( H \right)} \right]\,} \right)\,} \right\}\,$$

26 $$\vartheta = \frac{{L * J^{1} \left[ {G\left( H \right)} \right]}}{{\left\| L \right\| * \left\| {J^{1} \left[ {G\left( H \right)} \right]} \right\|}}$$

where, $\left( V \right)$ is the Zhonghua Distance between $\left( L \right)$ and $J^{1} \left[ {G\left( H \right)} \right]$, $\left( {r,\kappa } \right)$ are the uniform feature weight and Huber loss, respectively, and $\left( \vartheta \right)$ is the cosine similarity between $\left( L \right)$ and $J^{1} \left[ {G\left( H \right)} \right]$. Next, the gradients are clustered regarding $\left( L \right)$ and $\left( V \right)$ as,

27 $$X = L * \min \left\{ {\,V\left\langle {J^{1} \left[ {G\left( H \right)\,} \right]} \right\rangle \,} \right\}$$

28 $$X \to \left[ {X\left( {\left\langle {J^{1} \left[ {G\left( {H_{1} } \right)\,} \right]} \right\rangle + \left\langle {J^{1} \left[ {G\left( {H_{2} } \right)\,} \right]} \right\rangle + .. + \left\langle {J^{1} \left[ {G\left( {H_{n} } \right)\,} \right]} \right\rangle } \right)} \right]$$

Here, $\left( X \right)$ is the clustered output that indicates the aggregation of gradients from the heterogeneous hospitals, which is provided in Eq. (28). The cluster-wise aggregation continues until all the received gradients are clustered within the respective centroid. Further, the global prediction is done for analyzing the medical informatics aggregated from each local model.

#### Global prediction

As explained in Section "Prediction", the global CEP for each cluster is carried out based on $\left( X \right)$ using MCN-GNN. The clustered data is considered as a node matrix, and the CEP is performed. Let the attained global prediction be represented as $\left( Y \right)$. After attaining $\left( Y \right)$, the global update is done in the distributed hospitals.

### Distributed hospitals

The distributed hospitals in the FL system are those connected via the main server. Here, the $\left( n \right)$ number of heterogeneous hospitals is connected to the main server. Also, the prediction $\left( Y \right)$ obtained in the global model is updated to each local model.

#### Continual update

The updation of $\left( Y \right)$ into hospitals $\left( H \right)$ is performed using MEPDR. Regarding the avoidance of catastrophic forgetting, the Meta Experience Replay (MER) is utilized for continual updates. The MER stores a small buffer of past examples and mixes them with new data during update; hence, the model keeps “re-seeing” older tasks. Nevertheless, if replay samples are mostly from early tasks, then MER may overfit to those data and under-adapt to recent changes. Among various decay factors like Exponential Decay, Step Decay, and Polynomial Decay (PD) factor, the PD factor is selected because the Exponential and Step decay factors can cause abrupt learning-rate drops. PD diminishes the sampling probability, thereby ensuring that early-task samples don’t dominate the replay buffer and eliminating the overfitting issue. Further, by integrating the PD factor, the proposed MEPDR dynamically balances the influence of past and recent tasks, thereby achieving robust gradient updates across tasks and improving continual learning performance in a federated environment.

Initially, the gradient $\left( G \right)$ from the buffer samples of the local model and the new gradient $\left( Y \right)$ from the global model are collected. The input of MEPDR $\left( j \right)$ is expressed in Eq. (29) as,

29 $$j \to \left( {G,Y} \right)$$

Next, the PD $\left( \Re \right)$ that slows the decay of early samples is estimated to avoid the over-fitting issues and under-adaptation of the gradients by the local model.

30 $$\Re = \frac{1}{{\left[ {1 + \left( {\lambda * b} \right)} \right]\,^{\tau } }}$$

Here, $\left( \lambda \right)$ is the decay rate controlling factor, $\left( \tau \right)$ is the degree of polynomial decay that shapes the decay curve, and $\left( b \right)$ is the total iteration. Further, $\left( \Re \right)$ is utilized to blend $\left( G \right)$ and $\left( Y \right)$ and form a weighted gradient $\left( A \right)$.

31 $$A = \left( {\Re * G} \right) + \left[ {\left( {1 - \Re } \right) * Y} \right]$$

This $\left( A \right)$ is given to the authenticated local model $J^{1} \left[ {G\left( H \right)} \right]$, and the precise CEP is carried out in the local model.

32 $$A\mathop{\longrightarrow}\limits^{{{\mathrm{CEP}}}}J^{1} \left[ {G\left( H \right)} \right]$$

Hence, the local model gradients are updated regarding old data present in the local model itself and the new data attained from the global model of the FL.
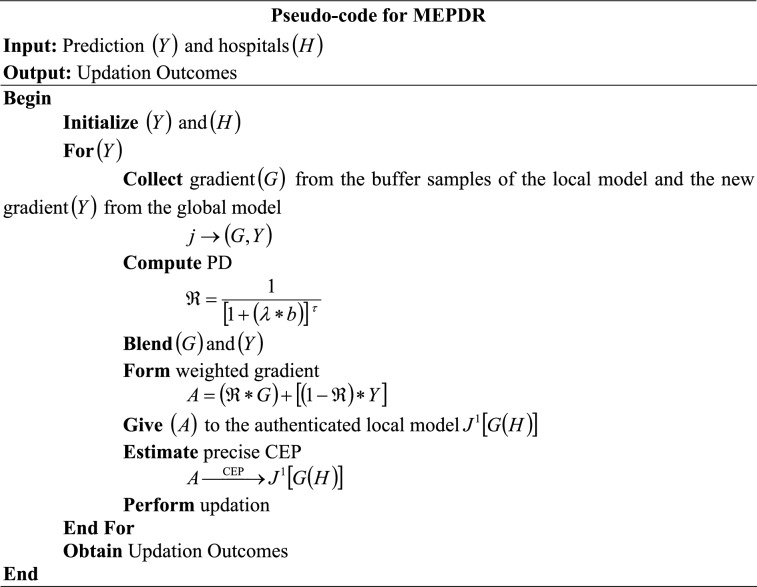


Thus, the catastrophic forgetting is mitigated using MEPDR, which ensures that the local model relies on both the local data and the incoming global gradient. Next, all the transactions are stored in the blockchain.

### Blockchain

Finally, the transactions $\left( C \right)$ of the hospitals $\left( H \right)$ regarding registration, login, local model CEP training, privacy preservation, hospital authentication, global aggregation, global prediction, and continual update are stored in the blockchain in the form of hash values $\left( \zeta \right)$. The blockchain is a distributed and immutable ledger technology, where every transaction is recorded in a block. Moreover, anyone in the network can verify the authenticity and order of updates without relying on a central authority.

33 $$C \to H\left( C \right) + \zeta$$

Thus, $\left( C \right)$ is recorded in the blockchain. Once $\left( C \right)$ is recorded, the updates can’t be modified or deleted without consensus from network participants. This improves the fairness and accountability of the proposed system. Hence, the proposed model predicted the client event and ensured security via FL. The proposed framework’s performance is explained in the subsequent section.

## Results and discussion

Here, the performance analysis as well as comparison of the proposed techniques are carried out to prove the proposed model’s effectiveness. The implementation is carried out on the working platform of PYTHON. Here, the experimental results are obtained by considering the average performance of the local models in the proposed federated learning scheme, illustrating that the proposed work is applicable for predicting diverse clinical events.

### Experimental setup

- Dataset Description

For the CEP in hospital 1, the “Heart Failure Prediction Dataset” that comprises the electronic health records of the patients is collected. Likewise, the “Stroke Prediction Dataset” is gathered from publicly available sources for the CEP in hospital 2. Also, for the CEP in hospital 3, the “Cirrhosis Prediction Dataset” is employed. Furthermore, the “Chronic Kidney Disease Dataset” is used for the CEP in hospital 4, whereas the “Diabetes Prediction Dataset” is applied for CEP in hospital 5. These dataset links are provided in the reference section. A total of 919 data are available to train the local model 1. Also, the “Stroke Prediction Dataset” consists of 5110 number of data to train the local model 2. Likewise, the “Cirrhosis Prediction Dataset” encompasses 418 numbers of data to train the local model 3. Similarly, “Chronic Kidney Disease Dataset” contains 400 numbers of data to train the local model 4, while the local model 5 is trained by using the “Diabetes Prediction Dataset”, which includes 100,000 numbers of data. The dataset specifications are provided in Table 1.

**Table 1:** Dataset specifications.

Dataset	Training (80%)	Testing (20%)	Total number of data
Heart Failure Prediction Dataset	735	184	919
Stroke Prediction Dataset	4088	1022	5110
Cirrhosis Prediction Dataset	334	84	418
Chronic Kidney Disease Dataset	320	80	400
Diabetes Prediction Dataset	80,000	20,000	100,000

From that, 80% and 20% of the data are used for training and testing, respectively.

(b) Hyperparameter Details

Next, the hyperparameters used for the proposed algorithms are given in Table 2.

**Table 2:** Hyperparameters for the proposed algorithms.

S.No	Parameters	Values
	Proposed MCN-GNN	
1	Optimizer	Adam
2	Initial learning rate	0.001
3	Activation function	ReLU
4	Number of GNN layers	2–4
5	Normalization method	MCN
6	Batch size	32
7	Number of training epochs	100
8	Loss function	Cross-entropy loss
	Proposed HRLSE	
1	Number of keys	4
2	Polynomial Modulus Degree	8192
3	Secret Key Size	256 bits
4	Ciphertext Size	~ 8–12 KB
5	Coefficient Modulus	[60, 40, 40, 60] bits
	TCG Construction	
1	Time window length	24–48 time steps
2	Temporal lag	1–3
3	Sliding window stride	1
4	Maximum temporal depth	5
5	Causality threshold	0.3–0.6
6	Minimum edge weight	0.05
7	Temporal smoothing factor	0.1–0.3
8	Edge pruning ratio	20–40%
	FL	
1	Federated rounds	100
2	Client participation ratio	0.3
3	Communication cost per round	84 MB
4	Uplink cost per round	72 MB
5	Downlink cost per round	12 MB
6	Mild Non-IID	0.5
7	Severe Non-IID	0.1
8	Data Size Sampling Variance	1.0
9	Client Sampling Ratio	0.3
10	Batch Size	32
11	Epochs per client	5
	Blockchain	
1	Blockchain type	Permissioned blockchain
2	Blockchain Platform	Hyperledger Fabric
3	Consensus Mechanism	Practical Byzantine Fault Tolerance (PBFT)
4	Network Participants	Hospitals, Client nodes, and Aggregation server
5	Peer Nodes	One for each hospital
6	Computational Overhead	Linear
7	Storage Overhead	Low
8	Gas Cost	0
9	Transaction Throughput	800–1200 tx/s
10	Storage per Transaction	0.5–1 KB

Table 2 displays the implementation details of the blockchain model. Here, the blockchain network had low computational overhead, low storage overhead, zero gas-cost, and high transaction throughput. Compared to other decentralized trust mechanisms (Decentralized Identifiers (DIDs) and Hashgraph), the blockchain network in the proposed model provided better auditability and storage due to its high trust level and validator selection.

(c) Mathematical Formulas

The mathematical formulas for the performance metrics used in the evaluation of the proposed method are depicted in Table 3.

**Table 3:** Mathematical formula for the performance metrics.

Performance metrics	Formula
Retained Accuracy	$\frac{1}{Tn - 1}\sum\limits_{s = 1}^{Tn - 1} {\alpha_{Tn,s} }$
Average Forgetting	$\frac{1}{Tn - 1}\sum\limits_{s = 1}^{Tn - 1} {\left( {\max_{v < s} \alpha_{v,s} - \alpha_{Tn,s} } \right)}$
Forward Transfer	$\frac{1}{Tn - 1}\sum\limits_{s = 2}^{Tn - 1} {\left( {\alpha_{s - 1,s} - \alpha_{0,s} } \right)}$
Backward Transfer	$\frac{1}{Tn - 1}\sum\limits_{s = 1}^{Tn - 1} {\left( {\alpha_{Tn,s} - \alpha_{s,s} } \right)}$
Accuracy	$\frac{TP + TN}{{TP + TN + FP + FN}}$
Precision	$\frac{TP}{{TP + FP}}$
Recall	$\frac{TP}{{TP + FN}}$
F-Measure	$2 * \frac{precision \cdot recall}{{precision + recall}}$
PPV	$\frac{TP}{{TP + FP}}$
NPV	$\frac{TN}{{TN + FN}}$
FNR	$\frac{FN}{{FN + TP}}$
FPR	$\frac{FP}{{FP + TN}}$
MSE	$\frac{1}{N}\sum\limits_{a = 1}^{N} {\left( {Y_{a} - \tilde{Y}_{a} } \right)^{2} }$
MAE	$\frac{1}{N}\sum\limits_{a = 1}^{N} {\left| {Y_{a} - \tilde{Y}_{a} } \right|}$
Loss Convergence Rate	$\frac{Loss\;value\;end - Loss\;value\;start}{{Communication\;rounds\;end - Communication\;rounds\;start}}$
Jain’s fairness index	$\frac{{\left( {\sum\limits_{a = 1}^{N} {Y_{a} } } \right)^{2} }}{{N\sum\limits_{a = 1}^{N} {Y_{a}^{2} } }}$
Security Level	$$\begin{gathered} \left( {W1 \times K\% } \right) + \left( {W2 \times C\% } \right) + \hfill \\ \left( {W3 \times E\% } \right) + \left( {W4 \times P\% } \right) + \hfill \\ \left( {W5 \times I\% } \right) \hfill \\ \end{gathered}$$
Attack Level	$\frac{Number\;of\;successful\;attacks}{{Total\;number\;of\;attacks}}$
Encryption Time	$$\begin{gathered} Encryption\;end\;time - \hfill \\ \quad \quad Encryption\;start\;time \hfill \\ \end{gathered}$$
Decryption Time	$$\begin{gathered} Decryption\;end\;time - \hfill \\ \quad \quad Decryption\;start\;time \hfill \\ \end{gathered}$$
Signature Creation Time	$$\begin{gathered} Signature\;creation\;end\;time - \hfill \\ \quad \quad Signature\;creation\;start\;time \hfill \\ \end{gathered}$$
Signature Verification Time	$$\begin{gathered} Signature\;verification\;end\;time - \hfill \\ \quad \quad Signature\;verification\;start\;time \hfill \\ \end{gathered}$$
Clustering Time	$Clustering\;end\;time - Clustering\;start\;time$
Silhouette Score	$\frac{Int - Near}{{\max \left( {Int,Near} \right)}}$
Davies–Bouldin Index	$\frac{1}{nC}\sum\limits_{b = 1}^{nC} {\mathop {\max }\limits_{c \ne b} \left( {\frac{{Cu_{b} + Cu_{c} }}{{Sp_{bc} }}} \right)}$

Where, $TP$, $TN$, $FN$, and $FP$ specify the true positive, true negative, false negative, and false positive, correspondingly, $K$ specifies the key length contribution, $C$ defines the attack complexity contribution, $E$ denotes the encryption algorithm strength contribution, $P$ exemplifies the privacy preservation contribution, $I$ illustrates the implementation security contribution, $N$ specifies the number of samples, $Y_{a}$ denotes the actual value of the $a^{th}$ sample, $\tilde{Y}_{a}$ indicates the predicted value of the $a^{th}$ sample, $Int$ illustrates average intra-cluster distance, $Near$ defines nearest cluster distance, $Tn$ represents the number of tasks $\left( s \right)$, $\alpha_{v,s}$ implies the accuracy at task $s$ after learning task $v$, $nC$ indicates the number of formed clusters, $Cu_{b}$ and $Cu_{c}$ exemplify intra-cluster scatter of cluster $b$ and $c$, respectively, and $Sp_{bc}$ defines inter-cluster separation.

### Performance evaluation

In this phase, the performance validation of the proposed methods, such as MEPDR, MCN-GNN, HRLSE, ExPrDSA, and CHIZD-KMC, is demonstrated to show the trustworthiness of each proposed model.

**Fig. 3:**
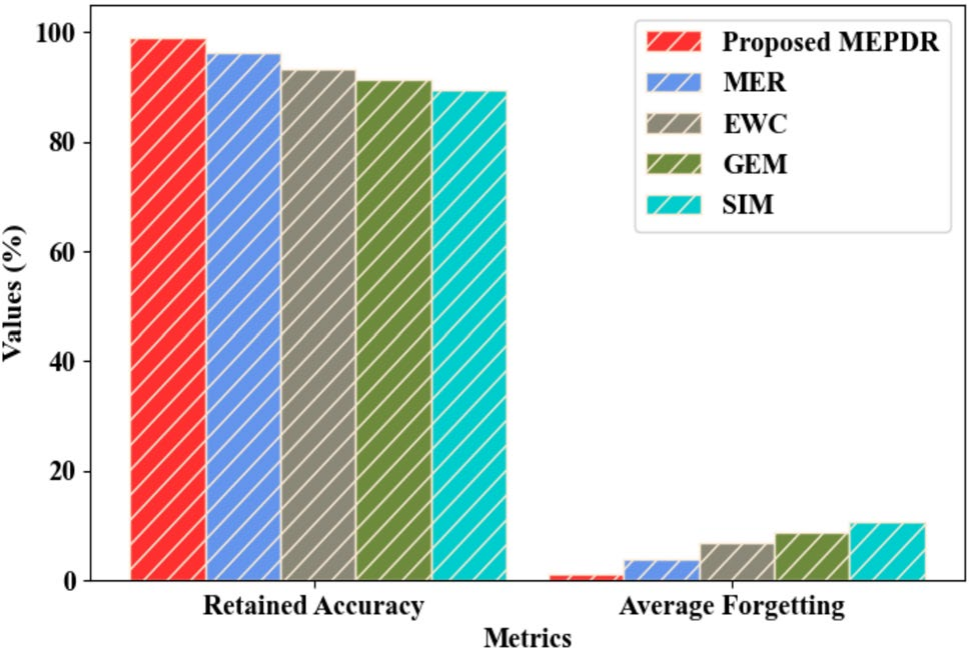
Comparative analysis of MEPDR.

Figure 3 illustrates the comparison of the proposed MEPDR and the prevailing methods like MER, Elastic Weight Consolidation (EWC), Gradient Episodic Memory (GEM), and Synaptic Intelligence Method (SIM) regarding continual update in the local model. The proposed MEPDR attained a retained accuracy of 98.95% and an average forgetting of 1.05%. However, the prevailing MER, EWC, GEM, and SIM attained retained accuracy values of 96.21%, 93.28%, 91.32%, and 89.47% and average forgetting values of 3.79%, 6.72%, 8.68%, and 10.53%, respectively. Thus, the integration of the gradient with the local model and the global update using PD improved the performance of the proposed method via continual update. PD excellently avoided the overfitting issue and under-adapted to recent changes. Therefore, the efficacy of the proposed methodology was proved.

**Fig. 4:**
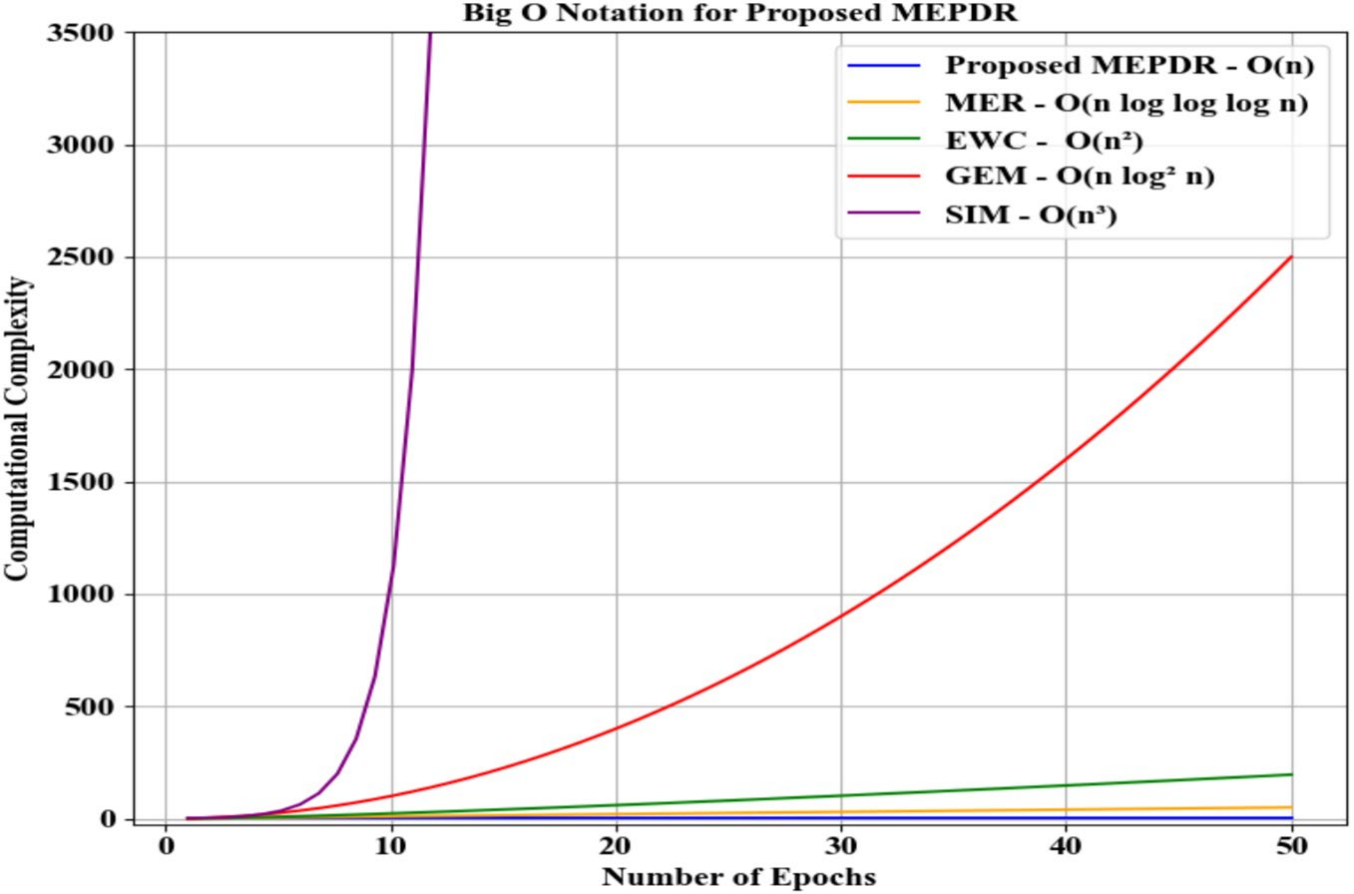
Computational complexity analysis.

Figure 4 depicts the computational complexity investigation (Big O) of the proposed MEPDR and conventional techniques. A computational cost measures the resources that an algorithm or task consumes to assess the efficiency. The proposed MEPDR had a high computational complexity of O(n) owing to the usage of PD. But, the prevailing MER, EWC, GEM, and SIM attained low computational complexities. Hence, the proposed model outperformed prevailing methods.

**Fig. 5:**
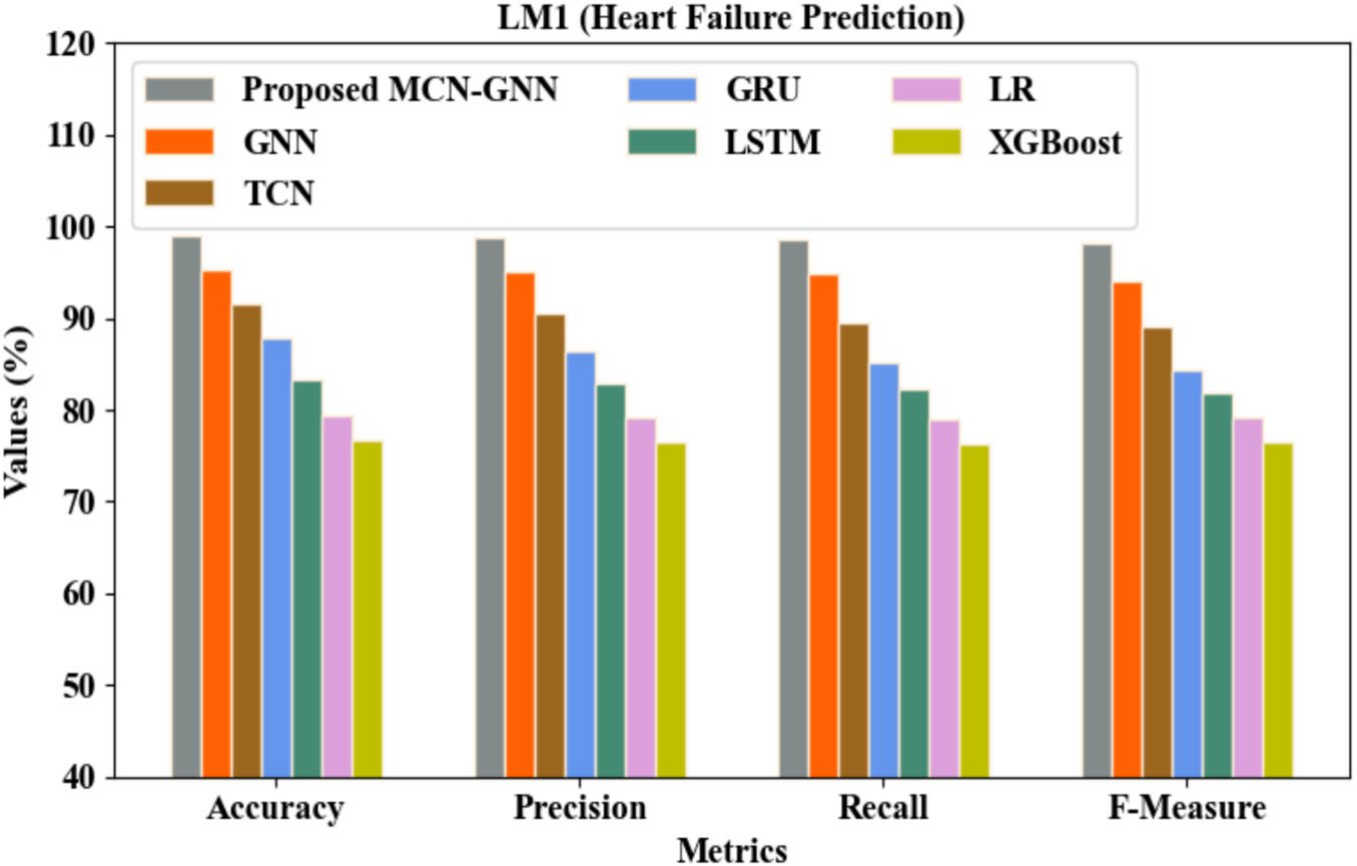
Graphical comparison for heart failure prediction.

The proposed classifier distinguished the complex nodes using the MCN, which effectively prevented the over-smoothing issue. Also, the features from the pre-processed electronic health record and the TCG were fed into the proposed MCN-GNN as a node matrix. As given in Table 4 and Fig. 5, heart failure was predicted in LM 1 with an accuracy of 98.97%, precision of 98.75%, recall of 98.62%, and F-Measure of 98.11%. But, the existing GNN, Temporal Convolutional Network (TCN), Gated Recurrent Unit (GRU), LSTM, Logistic Regression (LR), and eXtreme Gradient Boosting (XGBoost) achieved accuracy values of 95.32%, 91.65%, 87.91%, 83.28%, 79.34%, and 76.67%, precision values of 95.13%, 90.45%, 86.43%, 82.94%, 79.21%, and 76.53%, recall values of 94.83%, 89.47%, 85.15%, 82.38%, 79.02%, and 76.39%, and F-Measure values of 94.02%, 89.05%, 84.32%, 81.94%, 79.15%, and 76.46%, correspondingly. This showed that the proposed classifier predicted the heart failure event better than the existing classifiers.

**Table 4:** Comparative analysis for LM 1.

Techniques	Accuracy (%)	Precision (%)	Recall (%)	F-Measure (%)
Proposed MCN-GNN	98.97	98.75	98.62	98.11
GNN	95.32	95.13	94.83	94.02
TCN	91.65	90.45	89.47	89.05
GRU	87.91	86.43	85.15	84.32
LSTM	83.28	82.94	82.38	81.94
LR	79.34	79.21	79.02	79.15
XGBoost	76.67	76.53	76.39	76.46

**Fig. 6:**
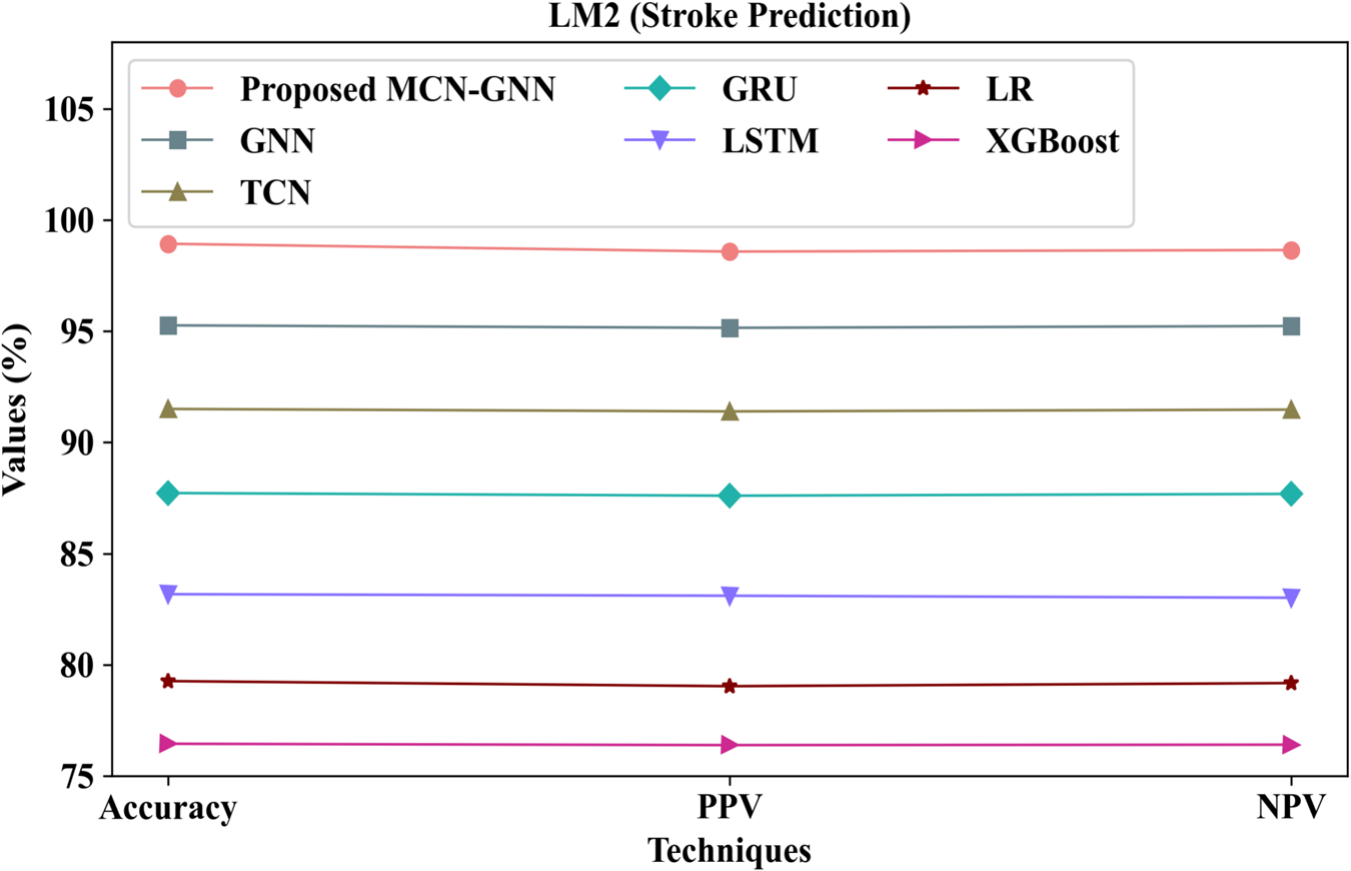
Pictorial representation for LM 2 regarding stroke prediction.

Figure 6 illustrates the pictorial representation of the proposed MCN-GNN and existing techniques concerning accuracy, Positive Predictive Value (PPV), and Negative Predictive Value (NPV). The proposed method attained high accuracy, PPV, and NPV of 98.92%, 98.57%, and 98.64% for stroke prediction in Local Model 2, respectively. However, the prevailing GNN and LSTM attained low accuracy values of 95.25% and 83.18%, correspondingly. Also, the prevailing GRU and LR obtained low PPVs of 87.61% and 79.04%, respectively. Likewise, the conventional TCN and XGBoost attained low NPVs of 91.46% and 76.41%, correspondingly. Here, the proposed model showed improved performance owing to the usage of MCN, which prevented over-smoothing and improved robustness in stroke prediction (LM 2). Thus, the effectiveness of the proposed method was proved.

**Fig. 7:**
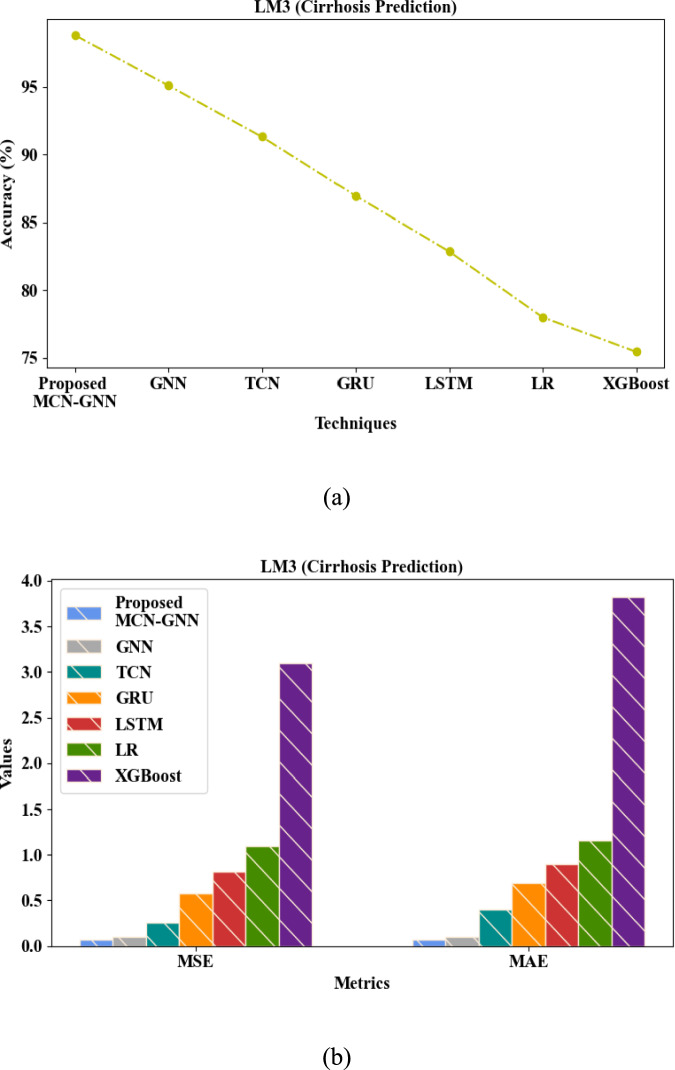
Performance evaluation of cirrhosis prediction for the proposed MCN-GNN in LM3 regarding (**a**) accuracy and (**b**) MSE and MAE.

Figure 7a and b depict the performance assessment of the proposed MCN-GNN and existing methodologies for LM 3 (cirrhosis prediction). Here, MCN was employed between the layers of GNN to prevent over-smoothing and reduce the extra feature averaging. For LM 3 (cirrhosis prediction), the proposed MCN-GNN achieved a high accuracy (98.83%) and low MSE (0.06471) and MAE (0.07164). But, the prevailing GNN, TCN, GRU, LSTM, LR, and XGBoost attained an average accuracy, MSE, and MAE of 84.955%, 0.989955, and 1.177543, respectively, which were poorer than the proposed method. Thus, the analysis proved that the proposed method accurately predicted the cirrhosis disease in LM 3.

**Fig. 8:**
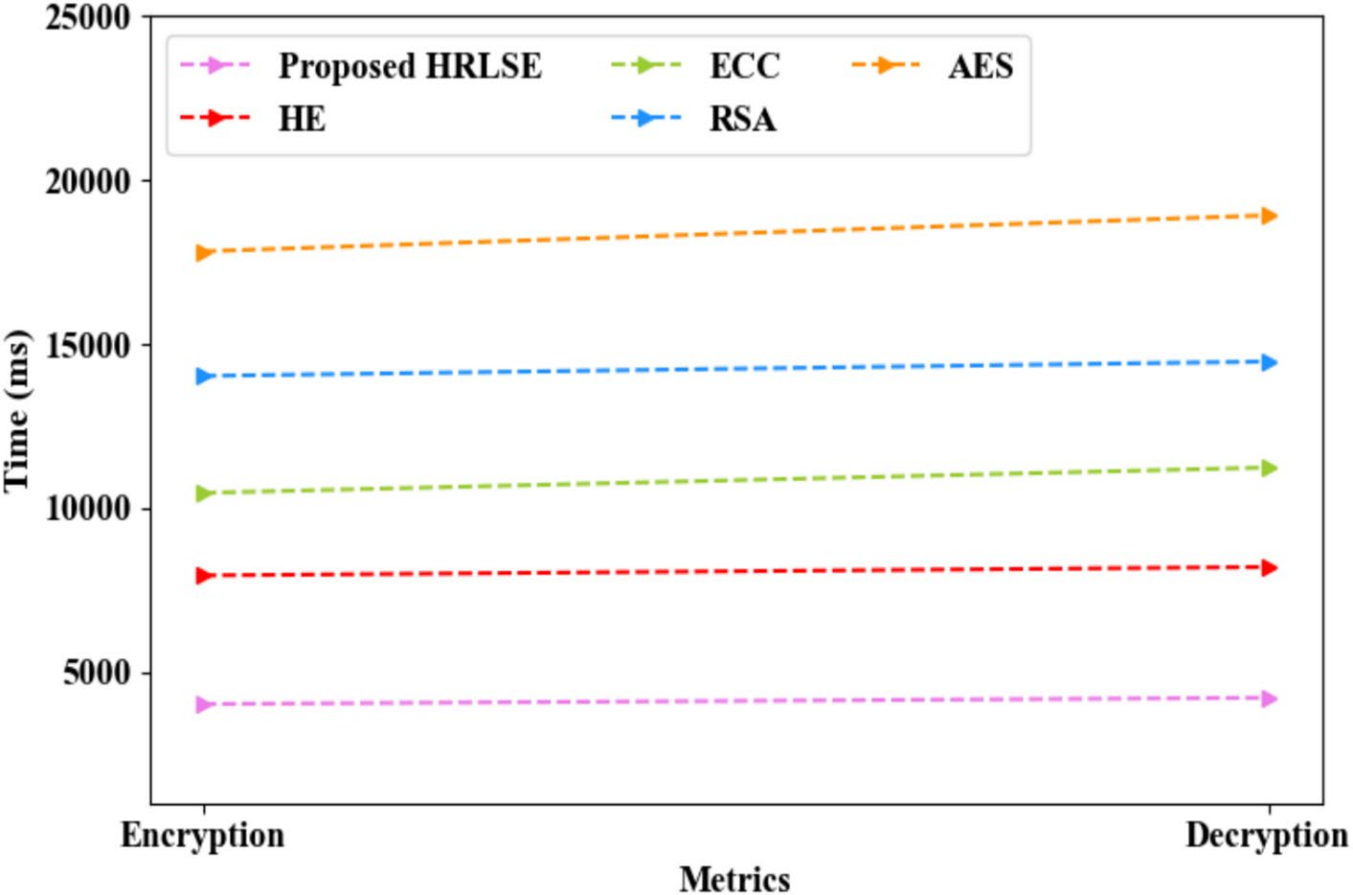
Graphical comparison of HRLSE.

The performance validation of the proposed HRLSE and the existing methodologies regarding privacy preservation of the local model’s gradients is depicted in Table 5 and Fig. 8. The proposed HRLSE and the traditional HE, Elliptic Curve Cryptography (ECC), Rivest-Shamir-Adleman (RSA), and Advanced Encryption Standard (AES) secured the local model’s gradient with security levels of 98.85%, 95.52%, 92.21%, 89.38%, and 88.04%, attack levels of 1.15%, 4.48%, 7.79%, 10.62%, and 11.96%, encryption times of 4021 ms, 7945 ms, 10463 ms, 14032 ms, and 17832 ms, and decryption times of 4214 ms, 8203 ms, 11237 ms, 14472 ms, and 18932 ms, correspondingly. In HRLSE, RLS effectively avoided the noise growth produced by continual homomorphic additions and multiplications. Hence, the avoidance of noise amplification using RLS in the proposed model enhanced the privacy preservation of the gradients compared to the conventional techniques.

**Table 5:** Comparative analysis of HRLSE.

Methods	Security level (%)	Attack level (%)	Encryption Time (ms)	Decryption time (ms)
Proposed HRLSE	98.85	1.15	4021	4214
HE	95.52	4.48	7945	8203
ECC	92.21	7.79	10,463	11,237
RSA	89.38	10.62	14,032	14,472
AES	88.04	11.96	17,832	18,932

**Fig. 9:**
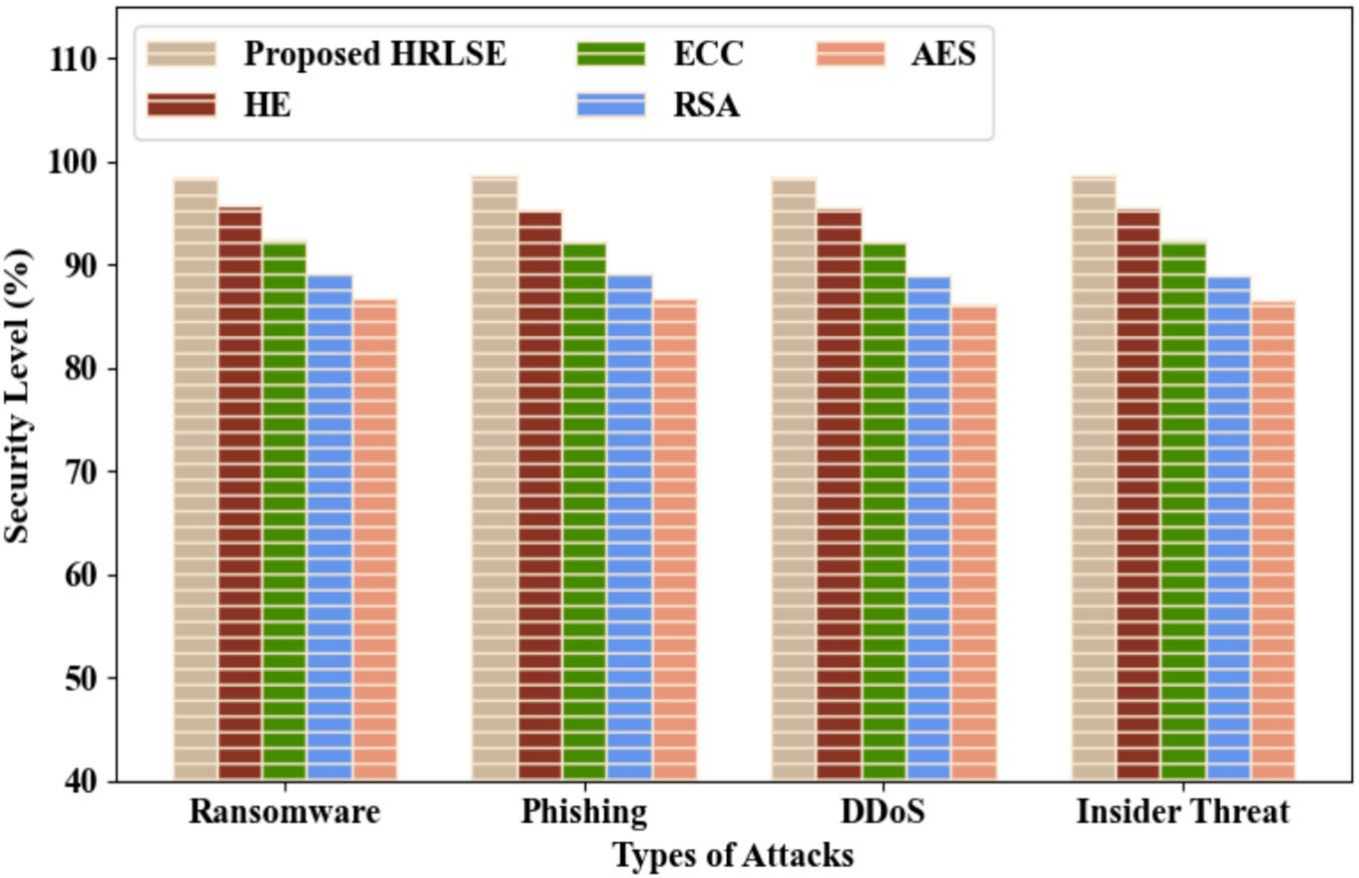
Security level evaluation for different types of attacks.

Figure 9 depicts the security level evaluation of the proposed HRLSE and traditional techniques for different types of attacks. Here, the proposed HRLSE employed RLS for avoiding the noise amplification. The proposed HRLSE achieved high security levels of 98.54%, 98.71%, 98.49%, and 98.69% for ransomware, phishing, Distributed Denial of Service (DDoS), and insider threat attacks, respectively. However, the prevailing HE, ECC, RSA, and AES achieved low average security levels of 91%, 90.86%, 90.71%, and 90.87% for different types of attacks, correspondingly. Thus, the proposed model attained improved performance across different attack scenarios.

**Fig. 10:**
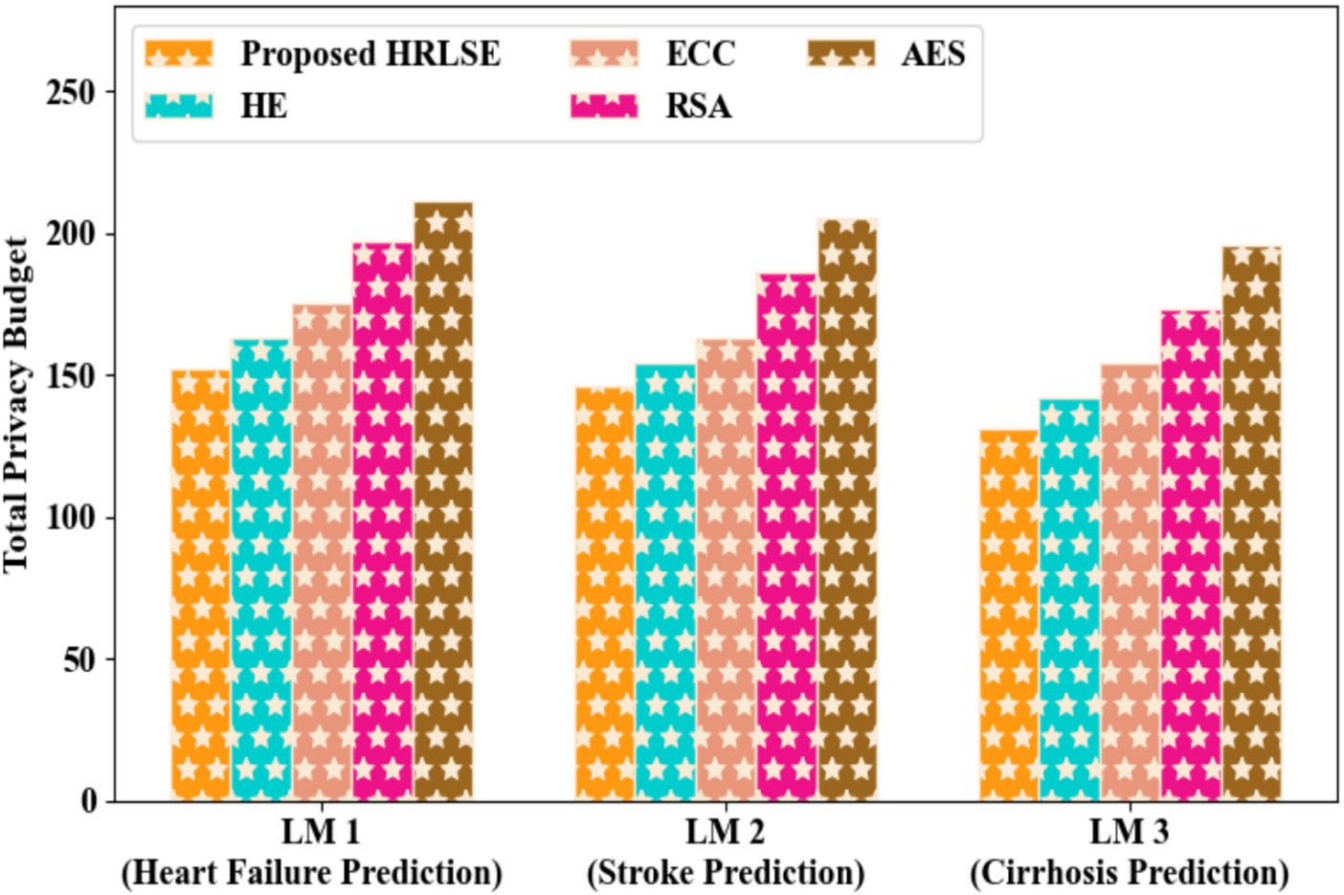
Differential privacy budget analysis of the proposed HRLSE.

From Fig. 10, it was proven that the proposed HRLSE effectively maintained a controlled differential privacy budget, ensuring that the local model gradients were securely encrypted while limiting the cumulative privacy loss. As a result, the proposed HRLSE demonstrated an appropriate and low privacy budget during model gradient privacy preservation. Therefore, the proposed HRLSE precisely protected model gradients without significantly affecting the model performance, making it appropriate for privacy-preserving federated learning in clinical environments.

**Fig. 11:**
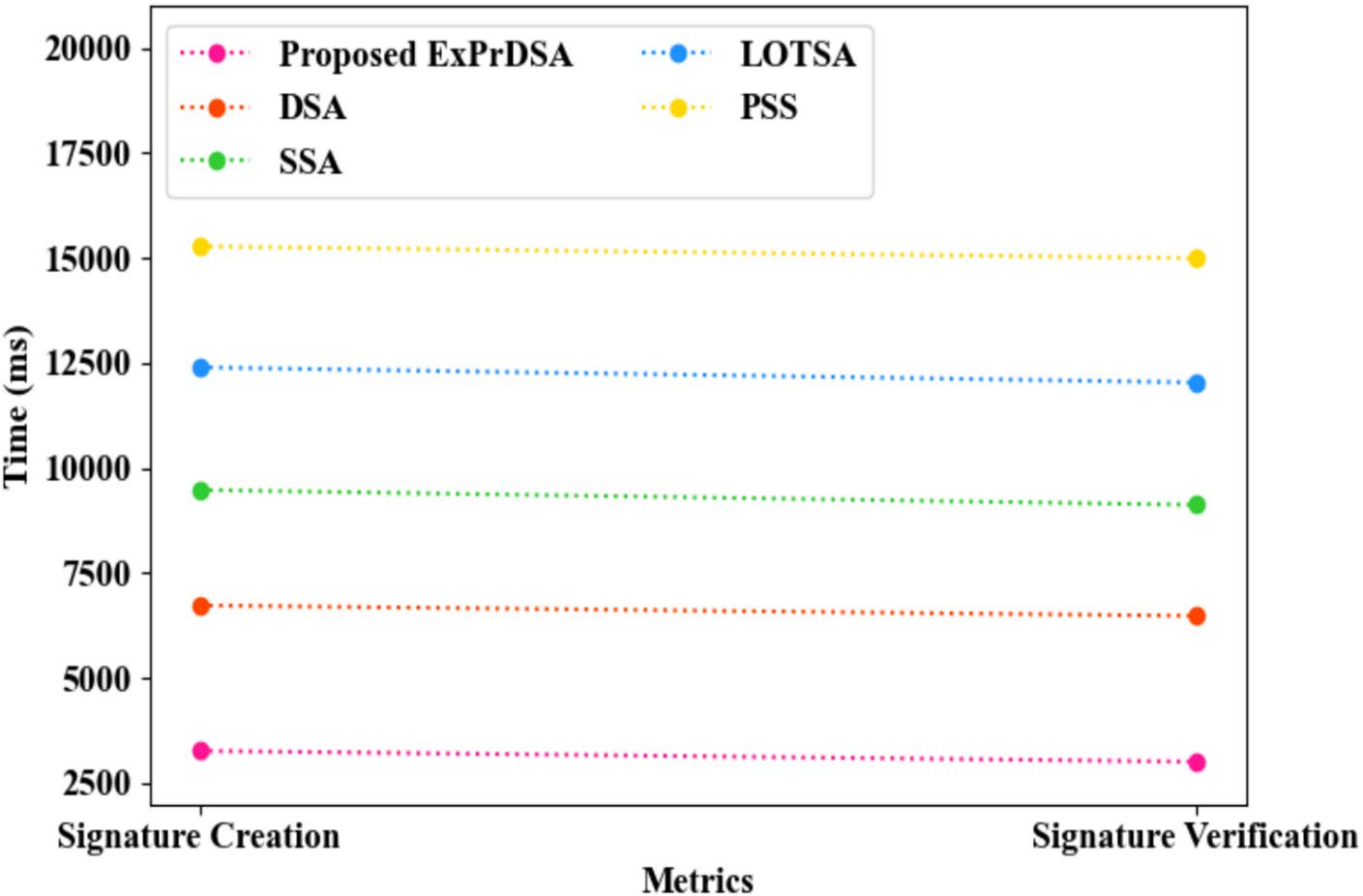
Graphical comparison of ExPrDSA.

The comparison of the proposed ExPrDSA and the prevailing methods regarding hospital authentication is illustrated in Fig. 11. The proposed model generated the hash function for digital signature creation using ExPr, which made collision prediction harder and improved the robustness against hash-flooding attacks. Thus, the hospital was authenticated with a Signature Creation Time (SCT) of 3271 ms and Signature Verification Time (SVT) of 3012 ms. However, the prevailing DSA, Schnorr Signature Algorithm (SSA), Lamport One-Time Signature Algorithm (LOTSA), and Probabilistic Signature Scheme (PSS) obtained SCTs of 6732 ms, 9478 ms, 12395 ms, and 15273 ms and SVTs of 6481 ms, 9123 ms, 12036 ms, and 14992 ms, respectively. Therefore, the outcomes depicted that the proposed ExPrDSA authenticated the hospitals better than the traditional methods.

**Fig. 12:**
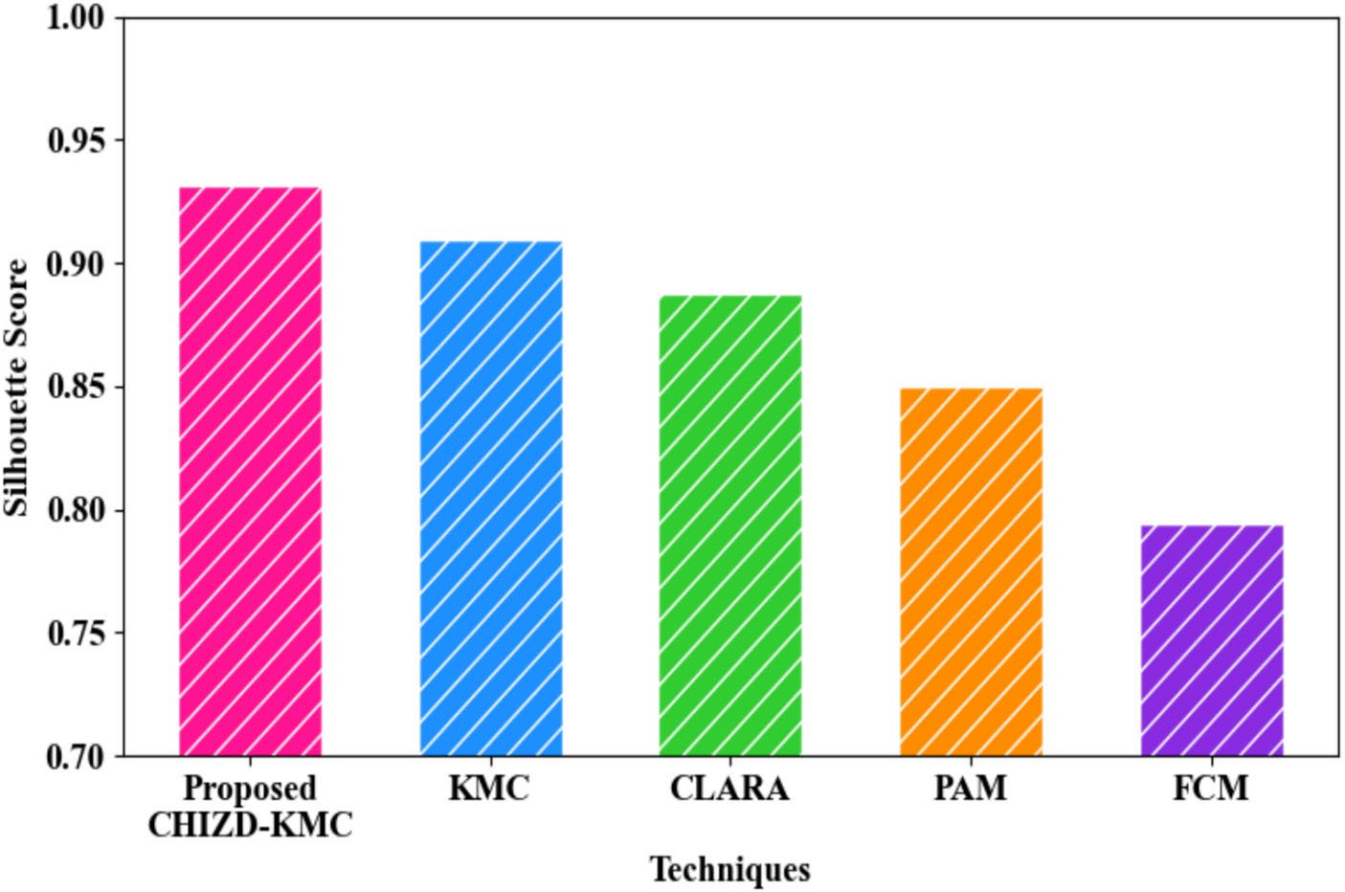
Graphical comparison regarding silhouette score.

The comparison of the proposed CHIZD-KMC and the traditional models regarding cluster-wise aggregation in the global model is given in Table 6 and Fig. 12. The proposed model aggregated the gradients with a clustering time of 4342 ms, a silhouette score of 0.9312, and a Davies–Bouldin Index (DBI) of 2.33. On the other hand, the prevailing KMC, Clustering Large Applications (CLARA), Partition Around Medoids (PAM), and Fuzzy C-Means (FCM) aggregated the gradients with clustering times of 8942 ms, 12382 ms, 16734 ms, and 20489 ms, silhouette scores of 0.9091, 0.8875, 0.8501, and 0.7945, and DBIs of 5.39, 9.94, 15.38, and 22.38, respectively. Thus, the generation of the centroid using CHI and the utilization of Zhonghua Distance in the proposed model enhanced the cluster-wise aggregation over existing models.

**Table 6:** Comparative analysis of CHIZD-KMC.

Techniques	Clustering time (ms)	Silhouette score	Davies–Bouldin index
Proposed CHIZD-KMC	4342	0.9312	2.33
KMC	8942	0.9091	5.39
CLARA	12,382	0.8875	9.94
PAM	16,734	0.8501	15.38
FCM	20,489	0.7945	22.38

Table 7 displays the performance assessment of the proposed Horizontal Federated Learning (HFL)-based CHIZD-KMC and prevailing FedDyn, SCAFFOLD, FedNova, FedProx, and FedAvg. Here, the proposed HFL-based CHIZD-KMC achieved a high loss convergence rate (− 0.035) and Jain’s fairness index (0.95), where HFL with CHI and ZD elevated the aggregation performance. However, the prevailing FedDyn, SCAFFOLD, and FedNova attained low loss convergence rates of − 0.029, − 0.026, and − 0.022, correspondingly. Also, the existing FedProx and FedAvg attained low Jain’s fairness index values of 0.71 and 0.63, respectively. The proposed model attained improved performance, showing the effectiveness of realistic multi-client FL. Thus, the proposed model excellently performed cluster-wise aggregation of the gradient related to different disease diagnosis outputs from various authenticated hospitals.

**Table 7:** Performance assessment for FL.

Techniques	Loss convergence rate	Jain’s fairness index
Proposed HFL-based CHIZD-KMC	− 0.035	0.95
FedDyn	− 0.029	0.89
SCAFFOLD	− 0.026	0.84
FedNova	− 0.022	0.76
FedProx	− 0.017	0.71
FedAvg	− 0.013	0.63

**Fig. 13:**
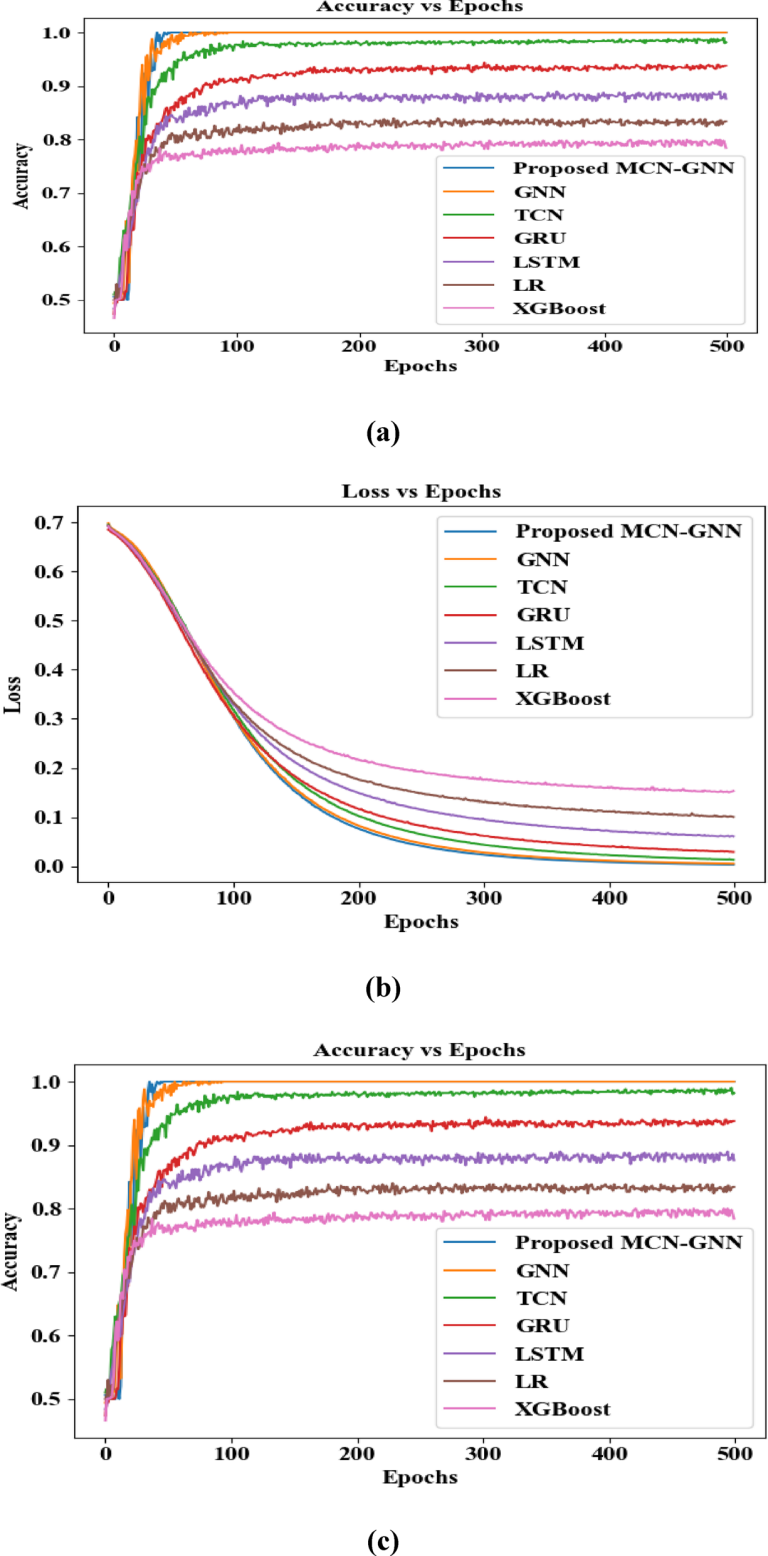
(**a**), (**b**), and (**c**): Performance analysis of the proposed MCN-GNN regarding Global Accuracy, Convergence Curves, and communication costs per-round/epochs.

As per Fig. 13a, b, and c, the proposed MCN-GNN achieved higher global accuracy, faster convergence, and lower communication cost per round during federated learning training. As a result, the proposed MCN-GNN consistently attained superior accuracy even at a higher number of communication rounds than the traditional methods. Similarly, the proposed MCN-GNN significantly reduced communication overhead at varying numbers of rounds, making it efficient for large-scale federated learning environments. Also, the proposed MCN-GNN demonstrated rapid and stable convergence across training rounds/epochs. Therefore, the proposed work ensured scalability in real-world clinical event prediction.

**Fig. 14:**
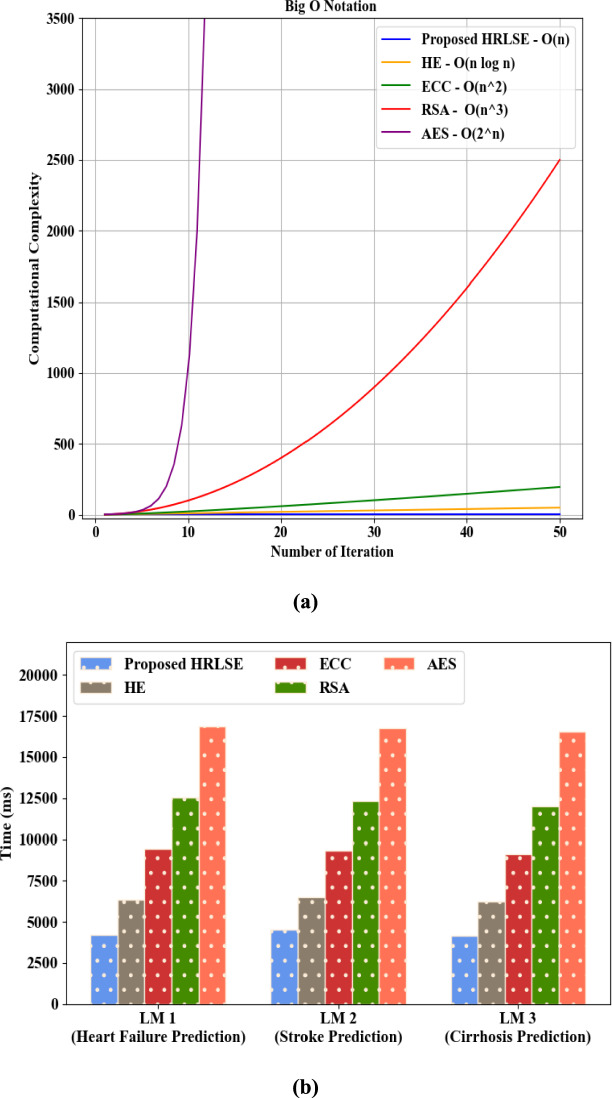
(**a**) and (**b**): Computational complexity and communication overhead analysis of the FL + HRLSE + Blockchain.

Figure 14a and b displayed that as the number of participating hospitals and training rounds increased, the computational complexity and communication overhead of the proposed FL + HRLSE + Blockchain framework remained manageable and scalable. Although the integration of security and blockchain introduced additional processing steps, the overall complexity increased in a controlled and non-linear manner, demonstrating that the framework was suitable for practical, large-scale federated learning deployments requiring excessive computational overhead.

### Empirical assessment of the local models (LM1, LM2, and LM3)

In this phase, the proposed framework’s effectiveness is validated under LM1, LM2, and LM3.

**Fig. 15:**
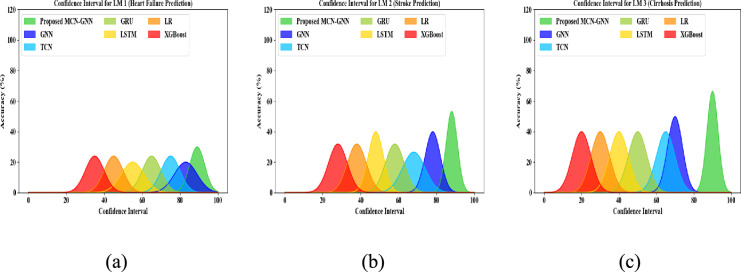
Confidence Interval Analysis for (**a**) LM1, (**b**) LM2, and (**c**) LM3.

Confidence interval analysis of the proposed MCN-GNN and conventional methods for LM1, LM2, and LM3 is depicted in Fig. 15. In general, a confidence interval visually indicates the statistical estimate that specifies the accuracy distributions across training epochs. It shows how the accuracy of the model differs with its peak value. Also, the confidence interval graph revealed an explicit uncertainty estimate by showing the prediction variability around accuracy. The proposed MCN-GNN had enhanced performance for CEP due to the usage of MCN-based normalization. But, the prevailing techniques attained limited performance.

**Fig. 16:**
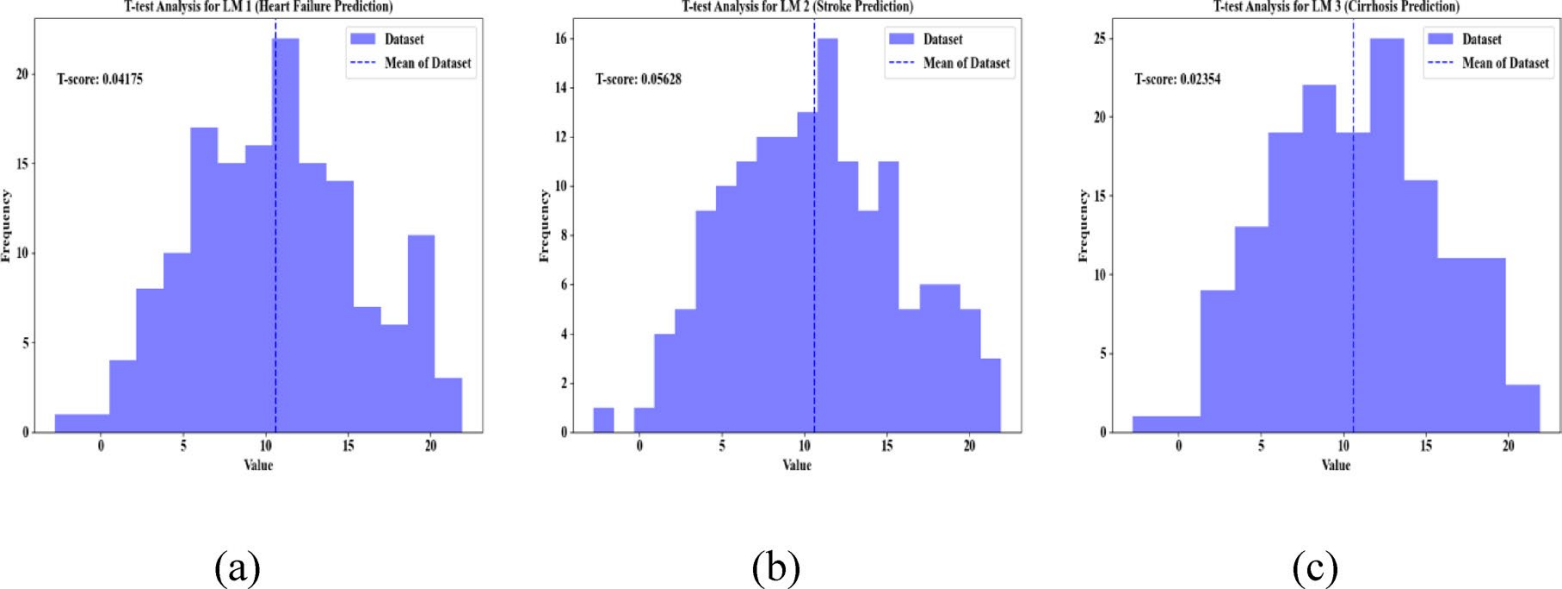
T-test validation for (**a**) LM1, (**b**) LM2, and (**c**) LM3.

Figure 16 shows the t-test validation for LM1, LM2, and LM3 of the proposed method. Here, based on the histogram of dataset values, the outcomes of a t-test validation are presented. The T-test measures the standardized difference betwixt the sample mean and the expected mean. In Fig. 16, the x-axis specifies the value range and the y-axis illustrates the frequency of observations. Also, the distribution of histogram values is displayed in the histogram bars. The reference point is marked by a dashed vertical line. Here, the proposed model has a t-test value of 0.04175, 0.05628, and 0.02354 for LM1, LM2, and LM3, respectively. Thus, the significance test shows the effectiveness of the proposed model.

**Fig. 17:**
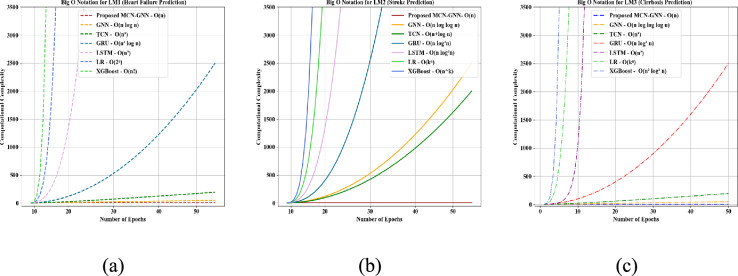
Big O complexity analysis of proposed MCN-GNN for (**a**) LM1, (**b**) LM2, and (**c**) LM3.

Big O complexity investigation of the proposed MCN-GNN and prevailing models for LM1, LM2, and LM3 is displayed in Fig. 17. Normally, Big O notation indicates the efficiency of an algorithm in time by showcasing its performance with input size. For LM1, LM2, and LM3, the proposed MCN-GNN achieved a low computational complexity of O(n) owing to the usage of MCN. Yet, the prevailing GNN, TCN, GRU, LSTM, LR, and XGBoost attained high computational complexity for all local models. Hence, the proposed model was better than the existing methodologies.

**Fig. 18:**
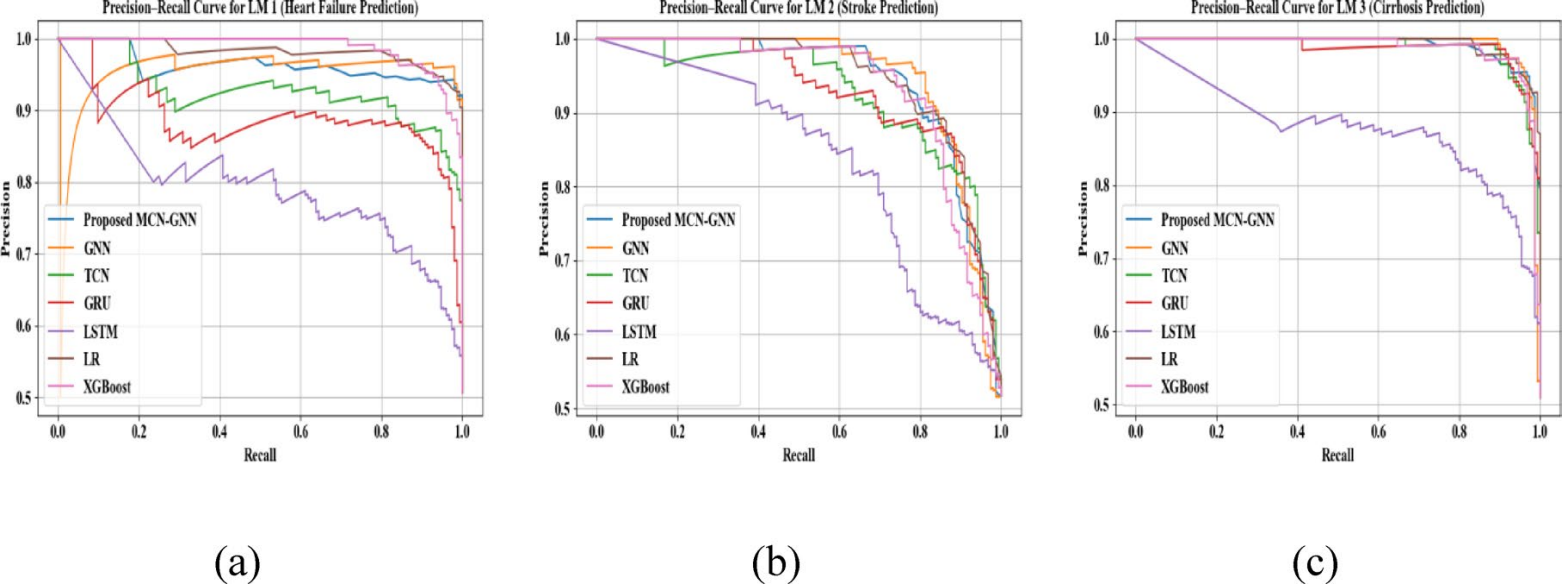
Precision recall curve analysis for (**a**) LM1, (**b**) LM2, and (**c**) LM3.

Figure 18 displays the precision-recall curve investigation of the proposed MCN-GNN and conventional models for LM1, LM2, and LM3. Here, the PR curve exemplifies how the precision and recall change at each step. A higher precision-recall curve specifies a strong and reliable prediction performance. Thus, the analysis showed that the proposed model was superior to the existing methods. Also, the MCN-based normalization was employed between the layers of GNN, thus preventing the over-smoothing issue.

**Fig. 19:**
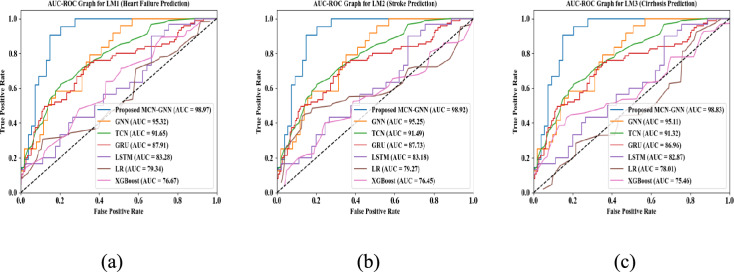
AUC-ROC curve validation for (**a**) LM1, (**b**) LM2, and (**c**) LM3.

Area Under the Curve-Receiver Operating Characteristics (AUC-ROC) analysis of the proposed model and prevailing techniques for LM1, LM2, and LM3 is displayed in Fig. 19. In the AUC-ROC curve, the higher values represent that the model superiorly predicted the diseases. The proposed model had higher AUC-ROC curve values owing to the usage of MCN. Thus, the reliability of the proposed model was demonstrated.

**Fig. 20:**
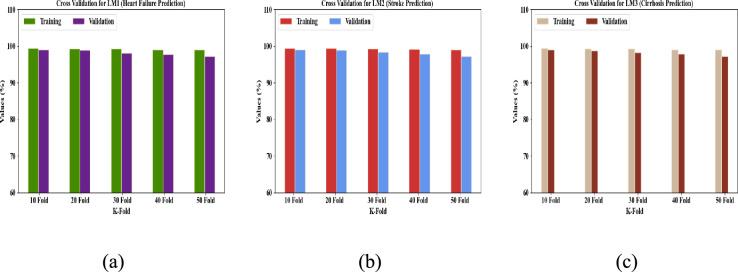
Cross-Validation analysis for (**a**) LM1, (**b**) LM2, and (**c**) LM3.

Figure 20 depicts the cross-validation investigation of the proposed method for LM1, LM2, and LM3. In general, cross-validation models the predictive value of a statistical model by dividing a dataset into training and testing sets, thus excellently evaluating the performance of the model. For LM1, the proposed model achieved training accuracies of 99.4587% (tenfold), 99.3568% (20 fold), 99.2874% (30 fold), 99.1025% (40 fold), and 99.0254% (50 fold), and validation accuracies of 99.1054% (tenfold), 98.97% (20 fold), 98.1254% (30 fold), 97.8423 (40 fold), and 97.2572% (50 fold), thus proving the effective performance trend across all K-fold splits. Also, the proposed model attained better accuracy regarding training and validation for LM2 and LM3. Overall, the cross-validation revealed the overfitting avoidance and generalizability of the proposed model.

**Fig. 21:**
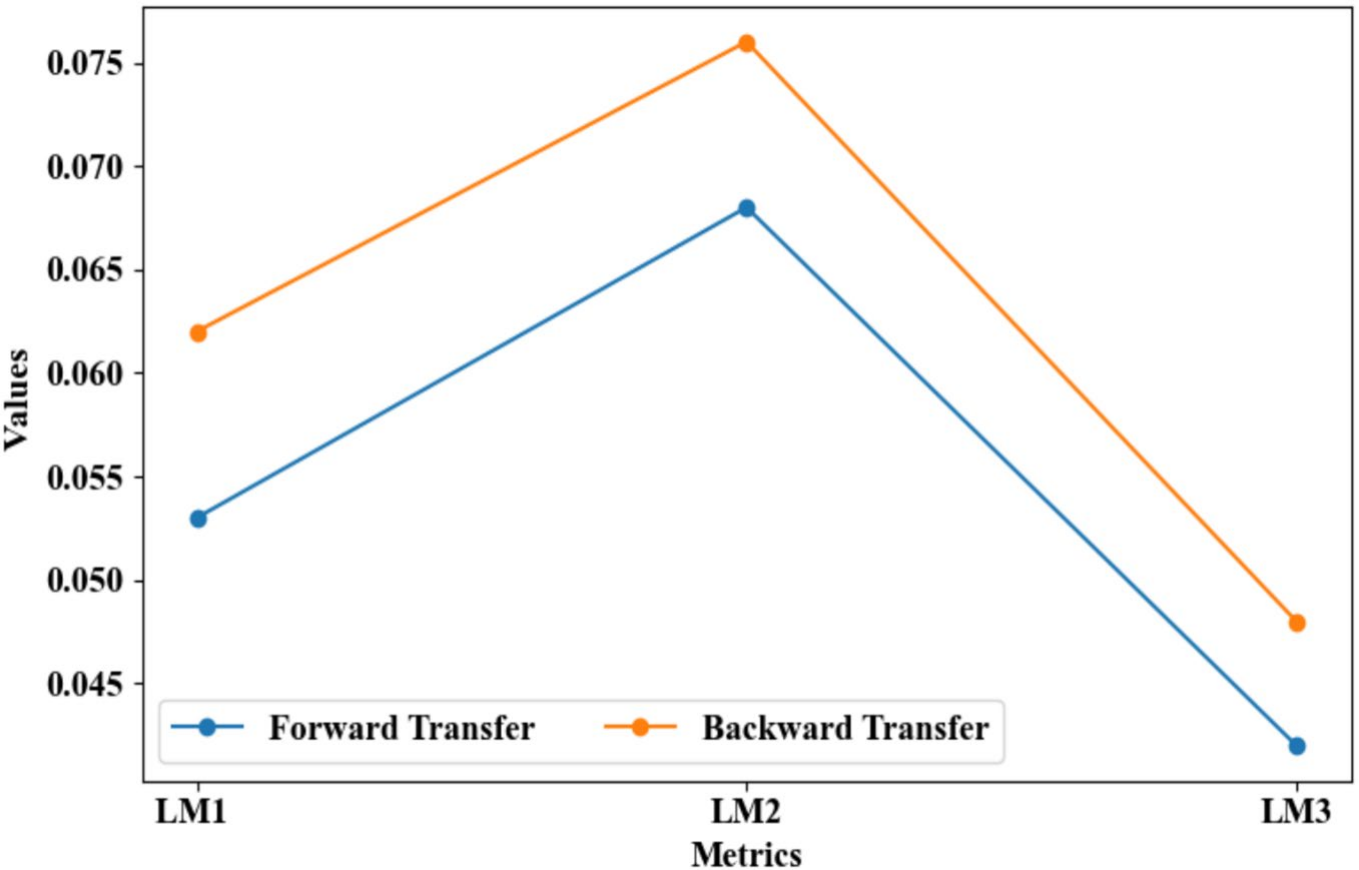
MEPDR evaluation on CL scenario with forgetting metrics.

Figure 21 displays the MEPDR evaluation of the proposed model on the CL scenario with respect to forgetting metrics like forward transfer and backward transfer. Here, the PD was modified in MEPDR to avoid the overfitting issues, thus improving the continual update performance. For local model 3, the proposed model had low Forward Transfer (0.042) and Backward Transfer (0.048), which were higher compared to other local models because LM3 consisted of a huge amount of training data. For local models 2 and 1, the proposed model had high forward transfer values of 0.068 and 0.053 and high backward transfer values of 0.076 and 0.062, correspondingly. Thus, the trustworthiness of the proposed model was demonstrated.

As per Table 8, the proposed models (LM1, LM2, and LM3) demonstrated high robustness under both evasion and poisoning attacks, achieving security levels above 98.8%. This achievement indicated that the proposed HRLSE effectively resisted the adversarial manipulations while preserving the integrity of model gradients and ensuring reliable learning even in hostile environments. Therefore, the proposed work showed significantly higher security levels across different local models against various adversarial threats.

**Table 8:** Robustness analysis of the proposed HRLSE under varying adversarial attacks regarding security level.

Proposed models	Adversarial attacks/security level (%)	
	Evasion attack	Poisoning attack
LM1	98.83	98.82
LM2	98.8	98.81
LM3	98.84	98.8

From Table 9, it was proven that the proposed models (LM1, LM2, and LM3) achieved high accuracy with very low error values, indicating strong calibration performance. Additionally, all their local models maintained higher accuracies and reduced errors, demonstrating that the predicted probabilities were well-aligned with the actual outcomes. Therefore, the proposed work produced accurate predictions while ensuring reliable and stable probability estimates in federated learning environments.

**Table 9:** Calibration analysis of the proposed models (LM1, LM2, and LM3).

Proposed models	Accuracy (%)	MSE	RMSE
LM1	98.97	0.985579	0.99276
LM2	98.92	0.978922	0.9894
LM3	98.83	0.989955	0.99496

### Ablation study analysis

Here, the ablation study is carried out for the proposed CHIZD-KMC.

Table 10 depicts the ablation study for the proposed CHIZD-KMC. Here, the proposed model with CHI and ZD attained a high silhouette score of 0.9312, where CHI superiorly initialized the centroids and ZD avoided suboptimal clustering. But, the proposed model without CHI attained a low silhouette score of 0.8957, whereas the proposed model without ZD obtained a very low silhouette score of 0.8671. Thus, the proposed CHIZD-KMC was better for cluster-wise aggregation in FL, which improved the FL convergence and stability.

**Table 10:** Ablation study for proposed CHIZD-KMC.

Techniques	Sihoutte score
Proposed model with CHI and ZD	0.9312
Proposed model without CHI	0.8957
Proposed model without ZD	0.8671

### Subjective assessment

In this phase, the proposed framework’s qualitative test assessment is done under various subjects during clinical event prediction.

Table 11 displays the qualitative test assessment for the proposed model in multiple evaluation scenarios and operational conditions. This validation is done for measuring the perceptual quality of the proposed model. Here, the feedback is provided in four scales, like 4 (excellent), 3 (good), 2 (moderate), and 1 (poor), for assessing the model’s performance. According to different evaluation scenarios and operational conditions, the model’s reliability, privacy assurance, computational overhead, and system usability show the robustness of the model.

**Table 11:** Qualitative test assessment.

Subjects	Evaluation scenario	Operational condition	Model reliability	Privacy assurance	Computational overhead	System usability
Subject 1	Multi-hospital collaboration	Normal	4	4	3	4
Subject 2	Non-IID Data Distribution	Heterogeneous patient data	3	4	3	3
Subject 3	Continual data updation	More clinical updates	4	3	2	4
Subject 4	Threat Environment	Adversarial update attempts	3	4	2	3

### Generalizability analysis through LM4 and LM5

Here, the proposed work is evaluated under LM4 and LM5 to enhance the model’s reliability in diverse clinical event prediction.

From Table 12, it was proven that the proposed MCN-GNN effectively performed clinical event prediction using the chronic kidney disease dataset compared to traditional models. As a result, the proposed MCN-GNN achieved 98.96% accuracy, 98.74% precision, 98.63% recall, and 98.1% f-measure, demonstrating its strong ability to accurately identify and classify clinical events. However, the traditional methods, such as GNN, TCN, GRU, LSTM, LR, and XGBoost, showed comparatively lower performance in chronic kidney disease prediction due to the limited ability to capture high-order temporal-causal dependencies. Thus, by incorporating the MCN with traditional GNN, the proposed work enabled robust feature representation and improved predictive reliability in distributed clinical environments.

**Table 12:** Performance analysis of the proposed work under chronic kidney disease dataset (LM4).

Techniques	Accuracy (%)	Precision (%)	Recall (%)	F-measure (%)
Proposed MCN-GNN	98.96	98.74	98.63	98.1
GNN	95.31	95.12	94.84	94
TCN	91.5	90.5	89.46	89
GRU	87.92	86.43	85.16	84.3
LSTM	83.29	82.95	82.39	81.95
LR	79.35	79.22	79.03	79.16
XGBoost	76.68	76.534	76.4	76.47

As per Table 13, the traditional methods demonstrated comparatively lower performance in clinical event prediction using the diabetic prediction dataset than the proposed MCN-GNN. For instance, the traditional GNN attained very low accuracy (95.31%), precision (95.12%), recall (94.82%), and f-measure (94.01%) due to the over-smoothing issue and limited capability to model long-range temporal-causal dependencies in heterogeneous EHR data. In contrast, the proposed MCN-GNN demonstrated superior performance by attaining higher accuracy, precision, recall, and f-measure values of 98.98%, 98.76%, 98.63%, and 98.12%, respectively. This depicted reliable and generalizable clinical event prediction in federated and heterogeneous healthcare environments.

**Table 13:** Effectiveness evaluation of the proposed work under the diabetic prediction dataset (LM5).

Techniques	Accuracy (%)	Precision (%)	Recall (%)	F-Measure (%)
Proposed MCN-GNN	98.98	98.76	98.63	98.12
GNN	95.31	95.12	94.82	94.01
TCN	91.64	90.44	89.46	89.06
GRU	87.90	86.42	85.14	84.33
LSTM	83.27	82.96	82.37	81.93
LR	79.33	79.22	79.03	79.16
XGBoost	76.66	76.54	76.38	76.45

### Comparative analysis

In this phase, the comparative analysis of the proposed work and traditional works is carried out.

Table 14 describes the comparison of the proposed system and the existing works concerning CEP. The existing works used classifiers, such as Harmo Self-Attentive Encoder (HarmoSATE), Blockchain-enabled Federated Learning (BFL), Multi-layer GNN, Residual learning-centric Deep Belief Network (RDBN), and Entropy Deep Belief Network (EDBN), for clinical event prediction. In the proposed framework, the electronic health record was pre-processed, and the TCG was constructed. Further, the FL-based MCN-GNN was utilized for clinical event prediction, thus attaining an accuracy of 98.97%, a recall of 98.62%, an F-Measure of 98.11%, and a precision of 98.75%. However, the existing^30–32^ predicted the client event without analyzing the cause and effect between the features, thereby attaining accuracy values of 74%, 92.6%, and 84.4%, respectively. Also, the prevailing^33,34^ couldn’t capture the temporal dependencies, thus attaining lower accuracy, recall, precision, and F-Measure values than the proposed work. When contrasted with the prevailing works, the proposed work improved the CEP.

**Table 14:** Existing works comparison.

Study	Methods	Accuracy (%)	Recall (%)	F-Measure (%)	Precision (%)
Proposed work	MCN-GNN	98.97	98.62	98.11	98.75
Lee et al.^30^	HarmoSATE	74	–	75	–
Ali et al.^31^	BFL	92.6	–	–	–
Tang et al.^32^	Multi-layer GNN	84.4	73.1	72.3	–
Markkandan et al.^33^	RDBN	92	91	92	–
Bhardwaj and Sumangali^34^	EDBN	95.07	96.54	95.98	95.44

### Clinical relevance evaluation

In real-world clinical settings, the proposed model offers clinical decision support by enabling heterogeneous hospitals to predict heart failure, stroke, and cirrhosis without sharing the raw patient data recorded in EHR. Here, the up-to-date evolving disease patterns are continually updated by the proposed model, thus supporting dynamic environments. Also, privacy-preserving gradient sharing provides early warning alerts and personalized care pathways. Likewise, the trust, traceability, and robustness against adversarial updates are ensured by the secure hospital authentication and blockchain-centric auditability. Overall, the proposed model is suitable for effective deployment in real-time distributed hospital networks, thereby enhancing the patient outcomes via a privacy-aware and continual learning CEP system.

## Conclusion

This paper proposed an effective framework for FL-centric CEP and privacy preservation across distributed hospitals. In the proposed work, the number of local models was trained using different datasets, such as the heart failure prediction dataset, stroke prediction dataset, and cirrhosis prediction dataset. Then, the trained local models were updated in the global model containing multiple hospitals. Here, clinical event prediction was done using MCN-GNN, attaining an accuracy of 98.97%. Then, the model’s gradients were preserved using HRLSE with a security level of 98.85%. Next, in the global model, the gradients were aggregated using CHIZD-KMC, thereby achieving an silhouette score of 0.9312. Here, the proposed HFL-based CHIZD-KMC achieved a high loss convergence rate (− 0.035) and Jain’s fairness index (0.95). Thereafter, the global prediction was carried out. Finally, the continual updation of the local model was done using MEPDR, attaining a retained accuracy of 98.95%. Meanwhile, all the transactions were stored in the blockchain for traceability. Hence, it was concluded that the proposed system efficiently predicted the client event, supported multi-client authentication, and preserved the gradients of EHR prediction using federated learning.

*Real World Performance:* By attaining high accuracy in CEP, the proposed model supports across heterogeneous hospitals without sharing raw EHR. Also, the continual learning provides continuous stability, thus making it appropriate for clinical environments. Likewise, robust privacy protection is ensured by the encryption technique. Similarly, auditability, security, and collaboration are ensured by the hospital authentication approach. Thus, the proposed model offers scalable, secure, and reliable deployment for real-world healthcare systems.

### Future scope

In the future, the communication between the global model and local model will be optimized to ensure real-time CEP integration into the hospital information system for critical event alerts.

Dataset Link: https://www.kaggle.com/datasets/fedesoriano/heart-failure-prediction; https://www.kaggle.com/datasets/fedesoriano/stroke-prediction-dataset; https://www.kaggle.com/datasets/fedesoriano/cirrhosis-prediction-dataset; https://www.kaggle.com/datasets/iammustafatz/diabetes-prediction-dataset/data; https://www.kaggle.com/datasets/mansoordaku/ckdisease/data.

## Supplementary Information

Supplementary Information.

## Data Availability

The datasets generated during and/or analyzed during the current study are available from publicly available sources.

## Code availability

The code developed for this study is provided as Supplementary Material along with this manuscript. It includes the implementations of the proposed models and algorithms used in the experiments. The shared code corresponds to the version used to generate the reported results and is sufficient for reproducing the study. The code is available for academic and research use.

## References

[1] Tajabadi, M., Martin, R. & Heider, D. Privacy-preserving decentralized learning methods for biomedical applications. *Comput. Struct. Biotechnol. J.* **23**, 3281–3287. 10.1016/j.csbj.2024.08.024 (2024). 39296807 10.1016/j.csbj.2024.08.024 PMC11408144

[2] Haripriya, R., Khare, N., Pandey, M. & Biswas, S. A privacy-enhanced framework for collaborative big data analysis in healthcare using adaptive federated learning aggregation. *J. Big Data.* **12**, 1–56. 10.1186/s40537-025-01169-8 (2025).

[3] Li, H. et al. Federated target trial emulation using distributed observational data for treatment effect estimation. *NPJ Digit. Med.* **8**, 1–15. 10.1038/s41746-025-01803-y (2025). 40593099 10.1038/s41746-025-01803-y PMC12214564

[4] Zwiers, L. C., Grobbee, D. E., Uijl, A. & Ong, D. S. Y. Federated learning as a smart tool for research on infectious diseases. *BMC Infect. Dis.* **24**, 1–14. 10.1186/s12879-024-10230-5 (2024). 39573994 10.1186/s12879-024-10230-5 PMC11580691

[5] Ali, M., Naeem, F., Tariq, M. & Kaddoum, G. Federated learning for privacy preservation in smart healthcare systems: A comprehensive survey. *IEEE J. Biomed. Health Inform.* **27**, 1–10. 10.1109/JBHI.2022.3181823 (2022). 10.1109/JBHI.2022.3181823 35696470

[6] Nasajpour, M. et al. Federated learning in smart healthcare: A survey of applications, challenges, and future directions. *Electronics* **14**, 1–40. 10.3390/electronics14091750 (2025).

[7] Oh, W. & Nadkarni, G. N. Federated learning in health care using structured medical data. *Adv. Kidney Dis. Health.* **30**, 1–27. 10.1053/j.akdh.2022.11.007 (2022). 36723280 10.1053/j.akdh.2022.11.007 PMC10208416

[8] Thakur, A. et al. Knowledge abstraction and filtering based federated learning over heterogeneous data views in healthcare. *NPJ Digit. Med.* **7**, 1–14. 10.1038/s41746-024-01272-9 (2024). 39414980 10.1038/s41746-024-01272-9 PMC11484763

[9] Nguyen, D. C. et al. Federated learning for internet of things: A comprehensive survey. *IEEE Commun. Surv. Tutor.* **23**, 1622–1658. 10.1109/COMST.2021.3075439 (2021).

[10] Murali, L., Gopakumar, G., Viswanathan, D. M. & Nedungadi, P. Towards electronic health record-based medical knowledge graph construction, completion, and applications: A literature study. *IEEE J. Biomed. Health Inform.* **143**, 1–13. 10.1016/j.jbi.2023.104403 (2023). 10.1016/j.jbi.2023.104403 37230406

[11] Javed, H., El-Sappagh, S. & Abuhmed, T. Robustness in deep learning models for medical diagnostics: Security and adversarial challenges towards robust AI applications. *Artif. Intell. Rev.* **58**, 1–107. 10.1007/s10462-024-11005-9 (2025).

[12] Xu, C., Qu, Y., Xiang, Y. & Gao, L. Asynchronous federated learning on heterogeneous devices: A survey. *Comput. Sci. Rev.* **50**, 1–15. 10.1016/j.cosrev.2023.100595 (2023).

[13] Madathil, N. T., Dankar, F. K., Gergely, M., Belkacem, A. N. & Alrabaee, S. Revolutionizing healthcare data analytics with federated learning: A comprehensive survey of applications, systems, and future directions. *Comput. Struct. Biotechnol. J.* **28**, 1–22. 10.1016/j.csbj.2025.06.009 (2025). 40606861 10.1016/j.csbj.2025.06.009 PMC12213103

[14] Xue, Z. et al. A resource-constrained and privacy-preserving edge-computing-enabled clinical decision system: A federated reinforcement learning approach. *IEEE Internet Things J.* **8**(11), 1–17. 10.1109/JIOT.2021.3057653 (2021).

[15] Zheng, Y. et al. A scoping review of self-supervised representation learning for clinical decision making using EHR categorical data. *NPJ Digit. Med.* **8**(1), 1–15. 10.1038/s41746-025-01692-1 (2025). 40517140 10.1038/s41746-025-01692-1 PMC12167381

[16] Meduri, K. et al. Leveraging federated learning for privacy-preserving analysis of multi-institutional electronic health records in rare disease research. *J. Econ. Technol.* **3**, 177–189. 10.1016/j.ject.2024.11.001 (2025).

[17] Messinis, S. C., Protonotarios, N. E. & Doulamis, N. Differentially private client selection and resource allocation in federated learning for medical applications using graph neural networks. *Sensors* **24**, 1–18. 10.3390/s24165142 (2024). 10.3390/s24165142 PMC11360477 39204839

[18] Ahmed, S., Kaiser, M. S., Chaki, S., Aloteibi, S. & Moni, M. A. Federated learning model with dynamic scoring-based client selection for diabetes diagnosis. *Knowl.-Based Syst.* **320**, 1–19. 10.1016/j.knosys.2025.113662 (2025).

[19] Nagamani, G. M. & Kumar, C. K. Design of an improved graph-based model for real-time anomaly detection in healthcare using hybrid CNN-LSTM and federated learning. *Heliyon* **10**, 1–22. 10.1016/j.heliyon.2024.e41071 (2024). 10.1016/j.heliyon.2024.e41071 PMC11696656 39759321

[20] Ali, A., Snášel, V. & Platoš, J. Health-FedNet: A privacy-preserving federated learning framework for scalable and secure healthcare analytics. *Res. Eng.* **27**, 1–34. 10.1016/j.rineng.2025.106484 (2025).

[21] Kuliha, M. & Verma, S. Secure internet of medical things based electronic health records scheme in trust decentralized loop federated learning consensus blockchain. *Int. J. Intell. Networks* **5**, 161–174. 10.1016/j.ijin.2024.03.001 (2024).

[22] Zhao, H., Sui, D., Wang, Y., Ma, L. & Wang, L. Privacy-preserving federated learning framework for multi-source electronic health records prognosis prediction. *Sensors* **25**, 1–15. 10.3390/s25082374 (2025). 10.3390/s25082374 PMC12031511 40285064

[23] Abaoud, M., Almuqrin, M. A. & Khan, M. F. Advancing federated learning through novel mechanism for privacy preservation in healthcare applications. *IEEE Access* **11**, 83562–83579. 10.1109/ACCESS.2023.3301162 (2023).

[24] Akter, M., Moustafa, N. & Turnbull, B. SPEI-FL: Serverless privacy edge intelligence-enabled federated learning in smart healthcare systems. *Cogn. Comput.* **16**, 2626–2641. 10.1007/s12559-024-10310-3 (2024).

[25] Edelson, M., Pham, A. & Kuo, T.-T. False-positive tolerant model misconduct mitigation in distributed federated learning on electronic health record data across clinical institutions. *Sci. Rep.* **15**, 1–11. 10.1038/s41598-025-04069-2 (2025). 40603924 10.1038/s41598-025-04069-2 PMC12223104

[26] Sharma, A., Sharma, A., Sharma, A., Sharma, A. & Guo, K. Intelligent medical diagnosis model based on graph neural networks for medical images. *CAAI Trans. Intell. Technol.* **10**, 1201–1216. 10.1049/cit2.70020 (2025).

[27] Bi, L. et al. FD-GATDR: A Federated-DeCentralized-learning graph attention network for doctor recommendation using EHR. *arXiv* 1–16; 10.48550/arxiv.2207.05750 (2022).

[28] Saemaldahr, R. & Ilyas, M. Patient-specific preictal pattern-aware epileptic seizure prediction with federated learning. *Sensors* **23**, 1–34. 10.3390/s23146578 (2023). 10.3390/s23146578 PMC10385318 37514873

[29] Mao, J. et al. Toward integrating federated learning with split learning via spatio-temporal graph framework for brain disease prediction. *IEEE Trans. Med. Imaging* **44**, 1–14. 10.1109/TMI.2024.3493195 (2025). 10.1109/TMI.2024.3493195 39509311

[30] Lee, T.-H., Kim, S., Lee, J. & Jun, C.-H. HarmoSATE: Harmonized embedding-based self-attentive encoder to improve accuracy of privacy-preserving federated predictive analysis. *Inf. Sci.* **662**, 1–22. 10.1016/j.ins.2024.120265 (2024).

[31] Ali, A. A. et al. Securing electronic health records using blockchain-enabled federated learning for IoT-based smart healthcare. *Clin. eHealth* **8**, 125–133. 10.1016/j.ceh.2025.04.002 (2025).

[32] Tang, T. et al. Personalized federated graph learning on Non-IID Electronic Health Records. *IEEE Trans. Neural Netw. Learn. Syst.* **35**, 1–15. 10.1109/TNNLS.2024.3370297 (2024). 10.1109/TNNLS.2024.3370297 38502617

[33] Markkandan, S., Bhavani, N. P. G. & Nath, S. S. A privacy-preserving expert system for collaborative medical diagnosis across multiple institutions using federated learning. *Sci. Rep.* **14**, 1–23. 10.1038/s41598-024-73334-7 (2024). 39333305 10.1038/s41598-024-73334-7 PMC11437101

[34] Bhardwaj, T. & Sumangali, K. An explainable federated blockchain framework with privacy-preserving AI optimization for securing healthcare data. *Sci. Rep.* **15**, 1–27. 10.1038/s41598-025-04083-4 (2025). 40595873 10.1038/s41598-025-04083-4 PMC12215731

