# Supplementary material for: Federated learning with continual update for privacy-preserving clinical event prediction across distributed hospitals using MCN-GNN
Source: Sci Rep. 2026 Mar 8;16:12608. doi: 10.1038/s41598-026-40964-y (PMC13087234; doi:10.1038/s41598-026-40964-y)
Supplement: Supplementary file 1 — Supplementary Information. [file 41598_2026_40964_MOESM1_ESM.docx]

**Prediction:** Mean-Centering Normalization-based Graph Neural Network (MCN-GNN)

import pandas as pd

import torch

from sklearn.neighbors import kneighbors_graph

from torch_geometric.data import Data

# -----------------------------

# Load dataset

# -----------------------------

df = pd.read_csv("disease_dataset.csv")

X = df.drop("label", axis=1).values

y = df["label"].values

# -----------------------------

# Mean-Centering Normalization

# -----------------------------

mean_vector = X.mean(axis=0)

X_mcn = X - mean_vector

x = torch.tensor(X_mcn, dtype=torch.float)

y = torch.tensor(y, dtype=torch.long)

# -----------------------------

# Build similarity graph

# -----------------------------

A = kneighbors_graph(X_mcn, n_neighbors=5, mode='connectivity')

edge_index = torch.tensor(A.nonzero(), dtype=torch.long)

data = Data(x=x, edge_index=edge_index, y=y)

import torch.nn.functional as F

from torch_geometric.nn import GCNConv

class MCN_GNN(torch.nn.Module):

def __init__(self, input_dim, hidden_dim, num_classes):

super().__init__()

self.conv1 = GCNConv(input_dim, hidden_dim)

self.conv2 = GCNConv(hidden_dim, num_classes)

def forward(self, data):

x, edge_index = data.x, data.edge_index

# Mean-centering inside model (optional research variant)

x = x - x.mean(dim=0)

x = self.conv1(x, edge_index)

x = F.relu(x)

x = self.conv2(x, edge_index)

return F.log_softmax(x, dim=1)

model = MCN_GNN(

input_dim=data.num_node_features,

hidden_dim=32,

num_classes=len(torch.unique(data.y))

)

optimizer = torch.optim.Adam(model.parameters(), lr=0.01)

for epoch in range(200):

model.train()

optimizer.zero_grad()

out = model(data)

loss = F.nll_loss(out, data.y)

loss.backward()

optimizer.step()

if epoch % 20 == 0:

print(f"Epoch {epoch}, Loss: {loss.item():.4f}")

**Model Gradient Privacy-Preservation:** Homomorphic Robust Log Scaling-based Encryption (HRLSE)

import numpy as np

import pandas as pd

from phe import paillier

# -----------------------------

# Robust Log Scaling

# -----------------------------

class RobustLogScaler:

def __init__(self, eps=1e-9):

self.eps = eps

self.median_ = None

self.iqr_ = None

def fit(self, X: np.ndarray):

self.median_ = np.median(X, axis=0)

q1 = np.percentile(X, 25, axis=0)

q3 = np.percentile(X, 75, axis=0)

self.iqr_ = (q3 - q1) + self.eps

return self

def transform(self, X: np.ndarray):

z = (X - self.median_) / self.iqr_

s = np.sign(z) * np.log1p(np.abs(z))

return s

def fit_transform(self, X: np.ndarray):

return self.fit(X).transform(X)

# -----------------------------

# HRLSE Encryptor

# -----------------------------

class HRLSEEncryptor:

def __init__(self):

self.public_key, self.private_key = paillier.generate_paillier_keypair()

def encrypt_vector(self, vec):

return [self.public_key.encrypt(float(v)) for v in vec]

def decrypt_vector(self, enc_vec):

return np.array([self.private_key.decrypt(v) for v in enc_vec], dtype=float)

# Homomorphic weighted sum: sum_i w_i * x_i (x_i encrypted)

def homomorphic_weighted_sum(self, enc_vec, weights):

assert len(enc_vec) == len(weights)

acc = enc_vec[0] * float(weights[0])

for i in range(1, len(enc_vec)):

acc = acc + enc_vec[i] * float(weights[i])

return acc

**Hospital Authentication:** Exponential Probing Digital Signature Algorithm (ExPrDSA)

import hashlib

import secrets

# -----------------------------

# Utilities

# -----------------------------

def H_int(*parts, q):

h = hashlib.sha256()

for p in parts:

if isinstance(p, int):

h.update(p.to_bytes((p.bit_length() + 7)//8 or 1, "big"))

elif isinstance(p, bytes):

h.update(p)

else:

h.update(str(p).encode())

return int.from_bytes(h.digest(), "big") % q

def modinv(a, m):

# Extended Euclid

r0, r1 = a % m, m

s0, s1 = 1, 0

while r1:

q = r0 // r1

r0, r1 = r1, r0 - q*r1

s0, s1 = s1, s0 - q*s1

if r0 != 1:

raise ValueError("No inverse")

return s0 % m

def powmod(a, e, m):

return pow(a, e, m)

# -----------------------------

# Domain parameters (toy-size for demo)

# Replace with proper safe primes for serious experiments.

# -----------------------------

# Small demo parameters (DO NOT use in production)

p = 0xE95E4A5F737059DC60DF5991D45029409E60FC09 # prime

q = 0xF518AA8781A8DF278ABA4E7D64B7CB9D49462353 # prime dividing p-1 in real setups

g = 2

# Ensure g^q mod p == 1 for real DSA groups. Here it's a demo.

# -----------------------------

# Key Generation

# -----------------------------

def keygen():

x = secrets.randbelow(q-1) + 1 # private

y = powmod(g, x, p) # public

return (x, y)

# -----------------------------

# Exponential Probing Nonce

# -----------------------------

def exp_probing_nonce(message: bytes, secret_seed: int, max_iters=32):

"""

k_i = H(m || seed || i)^(2^i) mod q, skip invalid values.

"""

for i in range(1, max_iters+1):

base = H_int(message, secret_seed, i, q=q)

if base == 0:

continue

exp = 1 << i # 2^i

k = powmod(base, exp, q)

if 0 < k < q:

return k

# fallback to random if probing fails

k = secrets.randbelow(q-1) + 1

return k

# -----------------------------

# Sign (ExPrDSA)

# -----------------------------

def sign(message: bytes, x_private: int, secret_seed=None):

if secret_seed is None:

secret_seed = secrets.randbits(256)

while True:

k = exp_probing_nonce(message, secret_seed)

r = powmod(g, k, p) % q

if r == 0:

continue

k_inv = modinv(k, q)

h = H_int(message, q=q)

s = (k_inv * (h + x_private * r)) % q

if s != 0:

return (r, s)

# -----------------------------

# Verify

# -----------------------------

def verify(message: bytes, signature, y_public: int):

r, s = signature

if not (0 < r < q and 0 < s < q):

return False

w = modinv(s, q)

h = H_int(message, q=q)

u1 = (h * w) % q

u2 = (r * w) % q

v = (powmod(g, u1, p) * powmod(y_public, u2, p) % p) % q

return v == r

# -----------------------------

# Example: sign MCN-GNN results / CSV bytes

# -----------------------------

if __name__ == "__main__":

x, y = keygen()

data = b"HRLSE-scaled features and MCN-GNN predictions"

sig = sign(data, x)

ok = verify(data, sig, y)

print("Signature:", sig)

print("Verified:", ok)

**Cluster-wise Aggregation:** Calinski–Harabasz Index with Zhonghua Distance-based K-Means Clustering (CHIZD-KMC)

import numpy as np

import pandas as pd

# -----------------------------

# Zhonghua Distance

# -----------------------------

class ZhonghuaDistance:

def __init__(self, X, lam=0.5, eps=1e-9):

self.std = X.std(axis=0) + eps

self.lam = lam

self.eps = eps

def pairwise(self, X, C):

# X: (n, d), C: (k, d)

diff = X[:, None, :] - C[None, :, :] # (n, k, d)

l1 = np.sum(np.abs(diff) / (self.std + self.eps), axis=2)

l2 = np.sum((diff**2) / (self.std**2 + self.eps), axis=2)

return l1 + self.lam * l2 # (n, k)

# -----------------------------

# K-Means with Zhonghua Distance

# -----------------------------

class ZD_KMeans:

def __init__(self, k=3, lam=0.5, max_iter=100, tol=1e-4, random_state=42):

self.k = k

self.lam = lam

self.max_iter = max_iter

self.tol = tol

self.random_state = random_state

def fit(self, X):

rng = np.random.default_rng(self.random_state)

n = X.shape[0]

# init centroids

idx = rng.choice(n, self.k, replace=False)

C = X[idx].copy()

dist = ZhonghuaDistance(X, lam=self.lam)

for _ in range(self.max_iter):

D = dist.pairwise(X, C) # (n, k)

labels = np.argmin(D, axis=1)

newC = np.vstack([

X[labels == i].mean(axis=0) if np.any(labels == i) else C[i]

for i in range(self.k)

])

if np.linalg.norm(newC - C) < self.tol:

break

C = newC

self.centroids_ = C

self.labels_ = labels

return self

# -----------------------------

# Calinski–Harabasz Index

# -----------------------------

def calinski_harabasz_index(X, labels, centroids):

n, d = X.shape

k = len(centroids)

overall_mean = X.mean(axis=0)

# Between-cluster dispersion

B = 0.0

for i in range(k):

Xi = X[labels == i]

ni = Xi.shape[0]

if ni == 0:

continue

diff = centroids[i] - overall_mean

B += ni * np.dot(diff, diff)

# Within-cluster dispersion

W = 0.0

for i in range(k):

Xi = X[labels == i]

if Xi.size == 0:

continue

diff = Xi - centroids[i]

W += np.sum(diff * diff)

return (B / (k - 1)) / (W / (n - k))

**Continual Update:** Meta Experience Polynomial Decay-based Replay (MEPDR)

import torch

import torch.nn as nn

import torch.optim as optim

import random

from copy import deepcopy

# -----------------------------

# Simple Model (replace with MCN-GNN later)

# -----------------------------

class SimpleNet(nn.Module):

def __init__(self, input_dim=10, num_classes=2):

super().__init__()

self.net = nn.Sequential(

nn.Linear(input_dim, 64),

nn.ReLU(),

nn.Linear(64, num_classes)

)

def forward(self, x):

return self.net(x)

# -----------------------------

# Replay Buffer

# -----------------------------

class ReplayBuffer:

def __init__(self, capacity=500):

self.capacity = capacity

self.buffer = []

def add(self, x, y):

if len(self.buffer) >= self.capacity:

self.buffer.pop(0)

self.buffer.append((x.detach().cpu(), y.detach().cpu()))

def sample(self, batch_size):

batch = random.sample(self.buffer, min(batch_size, len(self.buffer)))

xs, ys = zip(*batch)

return torch.stack(xs), torch.stack(ys)

# -----------------------------

# MER Trainer

# -----------------------------

class MERTrainer:

def __init__(self, model, lr=1e-3, beta=0.1, gamma=0.1, device="cpu"):

self.model = model.to(device)

self.opt = optim.Adam(self.model.parameters(), lr=lr)

self.criterion = nn.CrossEntropyLoss()

self.beta = beta # within-batch meta step

self.gamma = gamma # across-batch meta step

self.device = device

self.buffer = ReplayBuffer()

def train_step(self, x, y, replay_batch_size=16):

self.model.train()

x, y = x.to(self.device), y.to(self.device)

# Snapshot before batch (Reptile style)

theta_before = deepcopy(self.model.state_dict())

# Combine current + replay

if len(self.buffer.buffer) > 0:

rx, ry = self.buffer.sample(replay_batch_size)

x = torch.cat([x, rx.to(self.device)], dim=0)

y = torch.cat([y, ry.to(self.device)], dim=0)

# Shuffle combined batch

idx = torch.randperm(x.size(0))

x, y = x[idx], y[idx]

# Within-batch updates

for i in range(x.size(0)):

xi = x[i].unsqueeze(0)

yi = y[i].unsqueeze(0)

self.opt.zero_grad()

loss = self.criterion(self.model(xi), yi)

loss.backward()

self.opt.step()

# Within-batch meta update (β)

with torch.no_grad():

for p, p_before in zip(self.model.parameters(),

self._params_from_state(theta_before)):

p.data = p_before.data + self.beta * (p.data - p_before.data)

# Across-batch meta update (γ)

theta_after = self.model.state_dict()

with torch.no_grad():

for k in theta_before:

theta_after[k] = theta_before[k] + self.gamma * (theta_after[k] - theta_before[k])

self.model.load_state_dict(theta_after)

# Add current samples to buffer

for xi, yi in zip(x, y):

self.buffer.add(xi, yi)

def _params_from_state(self, state_dict):

for name, param in self.model.named_parameters():

yield state_dict[name]

**Temporal Casual Graph Construction:**

import networkx as nx

import matplotlib.pyplot as plt

class TemporalCausalGraph:

def __init__(self):

self.graph = nx.DiGraph()

def add_variable(self, var_name, time):

"""Add a node representing a variable at a specific time."""

node = (var_name, time)

self.graph.add_node(node)

def add_causal_edge(self, cause_var, cause_time, effect_var, effect_time, weight=1.0):

"""

Add a directed causal edge from cause to effect across time.

Example: X(t-1) -> Y(t)

"""

cause_node = (cause_var, cause_time)

effect_node = (effect_var, effect_time)

self.graph.add_node(cause_node)

self.graph.add_node(effect_node)

self.graph.add_edge(cause_node, effect_node, weight=weight)

def draw(self):

"""Visualize the temporal causal graph."""

pos = {}

# layout: time on x-axis, variables stacked on y-axis

variables = sorted({v for v, t in self.graph.nodes})

var_index = {v: i for i, v in enumerate(variables)}

for (var, time) in self.graph.nodes:

pos[(var, time)] = (time, -var_index[var])

labels = {node: f"{node[0]}(t={node[1]})" for node in self.graph.nodes}

nx.draw(self.graph, pos, with_labels=True, labels=labels, node_size=2000, font_size=8)

plt.show()
